# Structure of the agonist 12–HHT in its BLT2 receptor-bound state

**DOI:** 10.1038/s41598-020-59571-6

**Published:** 2020-02-14

**Authors:** Fabrice Giusti, Marina Casiraghi, Elodie Point, Marjorie Damian, Jutta Rieger, Christel Le Bon, Alexandre Pozza, Karine Moncoq, Jean-Louis Banères, Laurent J. Catoire

**Affiliations:** 1 Laboratoire de Biologie Physico-Chimique des Protéines Membranaires, UMR 7099, CNRS/Université de Paris, Institut de Biologie Physico–Chimique (FRC 550), 13 rue Pierre et Marie Curie, F–75005 Paris, France; 2 Present Address: Institut de Chimie Séparative de Marcoule, ICSM UMR 5257, Site de Marcoule, Bâtiment 426, BP 17171, F-30207 Bagnols sur Cèze Cedex, France; 3 Present Address: Department of Molecular and Cellular Physiology, Stanford University School of Medicine, 279 Campus Drive, 94305 Stanford California, USA; 4 Institut des Biomolécules Max Mousseron (IBMM), UMR 5247 CNRS, Université Montpellier, ENSCM, , 15 av. Charles Flahault, 34093 Montpellier, France; 5 Institut Parisien de Chimie Moléculaire, Sorbonne Université, CNRS, UMR 8232, Equipe Chimie des Polymères, 4 place Jussieu, 75252, Paris Cedex, 05 France

**Keywords:** Solution-state NMR, Solution-state NMR

## Abstract

G Protein-Coupled receptors represent the main communicating pathway for signals from the outside to the inside of most of eukaryotic cells. They define the largest family of integral membrane receptors at the surface of the cells and constitute the main target of the current drugs on the market. The low affinity leukotriene receptor BLT2 is a receptor involved in pro- and anti-inflammatory pathways and can be activated by various unsaturated fatty acid compounds. We present here the NMR structure of the agonist 12–HHT in its BLT2-bound state and a model of interaction of the ligand with the receptor based on a conformational homology modeling associated with docking simulations. Put into perspective with the data obtained with leukotriene B4, our results illuminate the ligand selectivity of BLT2 and may help define new molecules to modulate the activity of this receptor.

## Introduction

G protein-coupled receptors (GPCRs) are integral membrane proteins that allow the signal transduction from the outside to the inside of most of eukaryotic cells^1^. These receptors consist in a large family of proteins whose activities can be related to various ligands, from small organic compounds like neurotransmitters or hormones, to lipids, peptides or proteins. As such, they are key players in many biological processes and represent one of the most common target of clinical drugs^2,3^. Signal transduction through GPCRs concentrates a cascade of biological events that, if we exclude the constitutive activity, starts with the interaction of an extracellular signaling molecule with these membrane proteins, and triggers, at the end, a cellular response. This binding of a ligand onto its cognate receptor represents a fundamental stage in the activation process. To get a picture of this interaction at the atomic scale is not trivial as the number of high-quality crystals in the presence of natural agonists is limited^4–6^.

In addition to X-ray diffraction and cryo-electronic microscopy (cryo-EM)^7–10^, NMR spectroscopy can bring important information regarding conformational and energy landscapes^11–16^ or, as shown here, on the structure of natural GPCR ligands in their receptor bound-states. This technique can indeed provide a detailed description of the ligand in its bound-state, at physiological temperature and with a native protein^17–25^. Especially with very flexible ligands, like those described in this study, NMR data can constitute the basic input to subsequent X-ray- or cryo-EM-based molecular modeling of ligand/GPCR complexes.

The leukotriene receptors 1^26^ (BLT1) and 2^27–30^ (BLT2) are cell surface GPCRs that share 45% amino acid sequence identity in human and are involved in pro- and anti-inflammatory pathways^31–34^. They were initially named high (BLT1) and low leukotriene B4 (LTB4) (BLT2) receptors as the equilibrium dissociation constant (K_*d*_) values of LTB4 in the presence of membrane fractions transfected by either BLT1 or BLT2 is 20-fold weaker in the case of BLT2 compared to BLT1 transfected HEK 293 cells (*i*.*e*. ~1 nM and ~20 nM for BLT1 and BLT2, respectively)^30^. BLT1 receptor is essentially expressed in leukocytes and lymphocytes and is mainly activated by the LTB4^35^ which is a strong potent lipid inflammatory mediator. By contrast, BLT2 is expressed in various tissues and has been shown to bind to different arachidonic acid metabolites with moderate affinities, including LTB4^36^.

In 2008, the heptadecanoid 12S–hydroxyheptadeca-5Z,8E,10E-trienoic acid^37^ (12–HHT) was suggested to be the endogenous ligand of BLT2^38^. In membrane fractions of Chinese Hamster Ovary cells (CHO) transfected by BLT2, the half maximal inhibitory concentration (IC50) and the half maximal effective concentration (EC50) values of 12–HHT are about one order of magnitude lower than LTB4 while it does not bind to BLT1^38^. The main source of 12–HHT comes as a reaction product of the conversion of prostaglandin H2 to thromboxane A2 and malonyldialdehyde by the thromboxane synthase^39^. Recent studies highlighted an important activity of the 12–HHT/BLT2 axis in various pathologies, including inflammatory and allergic diseases^38,40–42^, wound healing^43^ and cancers^44–48^.

Here, we determined by NMR spectroscopy the three-dimensional (3D) structure of the agonist 12–HHT associated with human BLT2. As observed with the LTB4 in the presence of the same receptor^18^, 12–HHT adopts also a non-extended conformation. We propose also a tentative model of interaction of 12–HHT with BLT2 based on X-ray crystal structure conformational homology modeling and docking simulations, with the support of unequivocal experimental data.

## Methods

### Sample preparations

The heptdadecanoid 12–HHT and the eicosanoid LTB4 were obtained from Cayman Chemical, Ann Arbor, USA, as ethanolic solutions. The ethanol was extensively evaporated under vacuum. Then the eicosanoids in excess respectively to the receptor were directly dissolved by the NMR sample containing the receptor associated with perDAPol in a 100%-D_2_O solution (20 mM Tris/HCl buffer pH 8, 100 mM NaCl) at final concentrations of ~120 and ~140 *μ*M of 12–HHT and LTB4, respectively. The receptor concentration was ~15 *μ*M which gives rise to ligand/BLT2 molar ratio of 8 and ~9 for 12–HHT/BLT2 and LTB4/BLT2. Considering a percentage of properly folded receptor ranging from 50 to 70%^49^, this means an effective ligand/receptor ratios of 15 ± 4. Synthesis of perDAPol was performed as already described^50^ and the overexpression, purification and folding of perdeuterated human BLT2 receptor is detailed in Catoire *et al*.^18^.

### Site-directed mutagenesis

All mutations were introduced in the wild-type BLT2 receptor by PCR-mediated mutagenesis using the QuickChange multisite-directed mutagenesis kit (Stratagene) and the wild-type BLT2 construct as a template. Mutations were confirmed by nucleotide sequencing.

### Ligand binding assays

Agonist binding to the isolated BLT2 receptor was monitored through ligand-dependent receptor-catalyzed G protein activation, as described in Arcemisbéhère *et al*. (two different types of experiments were carried out to demonstrate 2010). G_*αi*2_ and G_*β*1*γ*2_ were prepared as previously described^51^. Briefly, agonist-dependent functional coupling of the purified receptor to G_*αi*2*β*1*γ*2_ was assessed through the rate of GTP*γ*S binding at increasing agonist concentrations determined by monitoring the relative increase in the intrinsic fluorescence (${\lambda }_{excitation}=300\,{\rm{nm}}$, ${\lambda }_{emission}=345\,{\rm{nm}}$) of G_*αi*2_ (200 nM of purified G protein) in the presence of BLT2 (20 nM) in buffer containing 10 mM MOPS, pH 7.2, 130 mM NaCl, and 2 mM MgCl_2_ at 15 °C after the addition of 10 *μ*M GTP*γ*S. The data were normalized to the fluorescence maximum obtained in the presence of saturating concentrations in 12–HHT (10 *μ*M).

### NMR spectroscopy

All NMR experiments were conducted at 25 °C and 700 MHz on a Bruker Avance spectrometer equipped with a cryoprobe. The dipolar interactions were detected and collected in a transferred mode, *i*.*e*. in the presence of an excess of ligand over the receptor thanks to a electrostatically-driven fast association and the perdeuteration of the receptor which allow detection of transferred cross-relaxation for GPCR ligands with equilibrium dissociation constants in the high-to-low nanomolar range (see the theoretical and experimental demonstration in Catoire *et al*.^19^). The following parameters were used for 2D NOESY experiments: 4 different mixing times (*τ*_*m*_ = 0.1 s, 0.2 s, 0.35 s, 0.5 s in the study of 12–HHT and *τ*_*m*_ = 0.05 s, 0.1 s, 0.2 s, 0.5 s with the LTB4); data size = 256(*t*_1_) × 8,192(*t*_2_) complex points, ${t}_{{1}_{{\max }}}=36.5$ ms, ${t}_{{2}_{max}}=585$ ms, 128 acquisitions per increment, experiment time = 11.5 to 15.7 hours. Water suppression was conducted by using an excitation sculpting scheme with gradients^52^. Prior to Fourier Transform, the time domain signal was apodized by a square cosine in both dimensions. No baseline correction was applied. ^1^H chemical shifts are referenced to H_2_O (calibrated at 4.7 ppm at 25 °C). Chemical shift assignments are based on COSY spectra from Catoire *et al*.^18^ and^19^. Data processing and analyzing were performed with TOPSPIN software.

### Structure calculations

12–HHT and LTB4 pdb files were produced with PRODRG^53^. Parameter and topology files were generated with XPLO2D (version 3.3.2)^54^. Structure calculations were performed with the program ARIA (Ambiguous Restraints for Iterative Assignment) (version 2.3)^55^ associated with CNS^56^ using standard protocols. For each ligand, calculations were based on four sets of NOE data corresponding to four distinct *τ*_*m*_ (see Tables S1–S4 and S6–S9 for 12–HHT and LTB4, respectively). A full relaxation matrix treatment of NOE data has been applied in ARIA/CNS to take into account indirect ^1^H-^1^H cross-relaxation pathways^57,58^. In the case of 12–HHT, only dipolar contacts involving H2, H3, and H17 with the other ligand protons were taken into account as these dipolar restraints display the lowest level of non-specific binding contribution to the peak volumes (unstructured parts in the ligand in the absence of the receptor). For LTB4, only protons at both ends interacting with the other protons in the ligands, *i*.*e*. H2, H3, H4 and H16, H17, H18, H19 and H20, were taken into account in the structure calculation. The structures were drawn using the software PyMOL.

### Homology modelling of receptors and ligand docking simulations

Homology modelling of BLT2 based on X-ray crystal structures was performed with the software Modeller (version 9.2)^59–61^. Several structures were tested, including the two mentionned in this manuscript: BLT1 (pdb code 5x33^62^) and *β*2AR (pdb code 3p0g^4^). Docking simulations of 12–HHT in human BLT2 receptor were subsequently performed with HADDOCK (version 2.2) taking as *active residues* S174^*ECL*2^ and R270^7.35^ only.

## Results

### Structure of 12–HHT associated with BLT2 receptor

**Figure 1:**
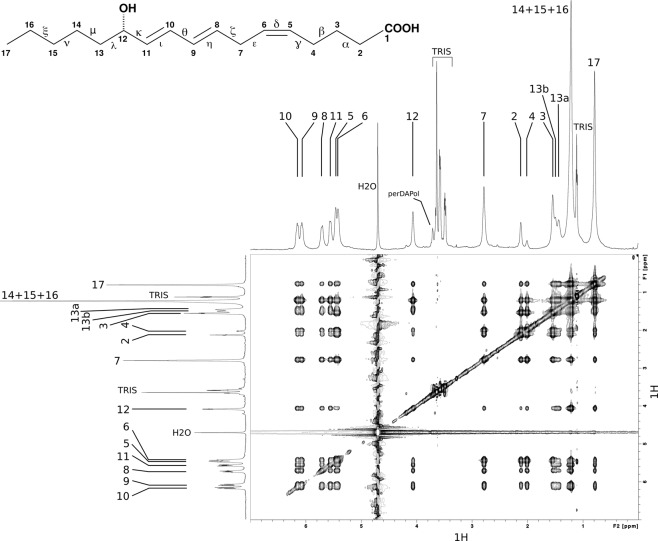
Dipolar interactions in the 12–HHT/u-^2^H-wtBLT2/perDAPol sample observed in a 2D NOESY spectrum (*τ*_*m*_ = 0.5 s, *ν*_*H*_ = 700 MHz, 25 °C, [12–HHT] = 120 *μ*M, [BLT2] = 15 *μ*M). The corresponding 1D ^1^H spectrum is shown above the 2D spectrum and a 1D spectrum of free 12–HHT in solution is displayed on the left side. Numbers refer to the protons annotated on the 12–HHT chemical structure indicated above the spectrum.

The NMR study of 12–HHT in its receptor-bound state was realized *in vitro* in a detergent-free solution^63^ following a method that has been already applied with the LTB4 in the presence of the same receptor^18,19^. Briefly, the heterologous human BLT2 receptor was expressed in *Escherichia coli* in a 100%-D_2_O solution to inclusion bodies^64,65^ and was subsequently folded to its native state using amphipols^49,66^. The NMR structure of 12–HHT is based on the detection of dipolar interactions in the ligand through two-dimensional homonuclear ^1^H Nuclear Overhauser Effect SpectroscopY (NOESY) experiments^67^. The dipolar interactions were collected in a transferred mode in the presence of an excess of ligand over the receptor. Indeed, it has been demonstrated that solution-state NMR can detect transferred NOEs even with equilibrium dissociation constants below the micromolar range because of *i*) an inherent ultra-fast diffusive association of these negatively charged agonists onto a highly positively charged extracellular surface, and *ii*) the slowing down of the ^1^H-^1^H cross-relaxation thanks to receptor perdeuteration^19^. In order to improve the number and quality of intra-ligand ^1^H-^1^H dipolar contacts, BLT2 was maintained soluble and stable in solution associated with a perdeuterated amphipol named perDAPol^50^. Compared to the pioneer study of LTB4 associated with BLT2, perDAPol offered the possibility to observe intra-aliphatic ^1^H dipolar interactions in the ligand (Fig. 1) (for a comparative observation, see Fig. S1).

In the presence of BLT2 associated with either amphipols or nanodiscs, 12–HHT displays a higher proportion of non-specific binding compared to LTB4 (see for instance Supplementary Fig. S8 in Casiraghi *et al*.^11^). This is presumably due to a more hydrophobic character which favours the interaction of the ligand with the belt of surfactant molecules or lipids^11^. To correctly assess the presence of specific intra-ligand dipolar interactions, the NMR collection of constraints was based on a rigorous observation of specific intra-ligand ^1^H-^1^H dipolar interactions in the bound-state in the presence of a perdeuterated receptor^18,19^. In the absence of the receptor, *i*.*e*. in the presence of perDAPol only, we observed the absence and/or the presence of weak ^1^H–^1^H dipolar interactions between aliphatic protons located at both ends, *i*.*e*. from protons H2 to H4 on one side, and from H13 to H17 on the other side, with the other ^1^H in the ligand (Figs. S2 and S3). This indicates that both ligand ends are not structured in the absence of the receptor. Moreover, non-specific ^1^H to ^1^H interactions between the ligand and the surfactant can be observed in a 2D NOESY spectrum (*e*.*g*. the regions squared with a green dashed line in Fig. S2).

**Figure 2:**
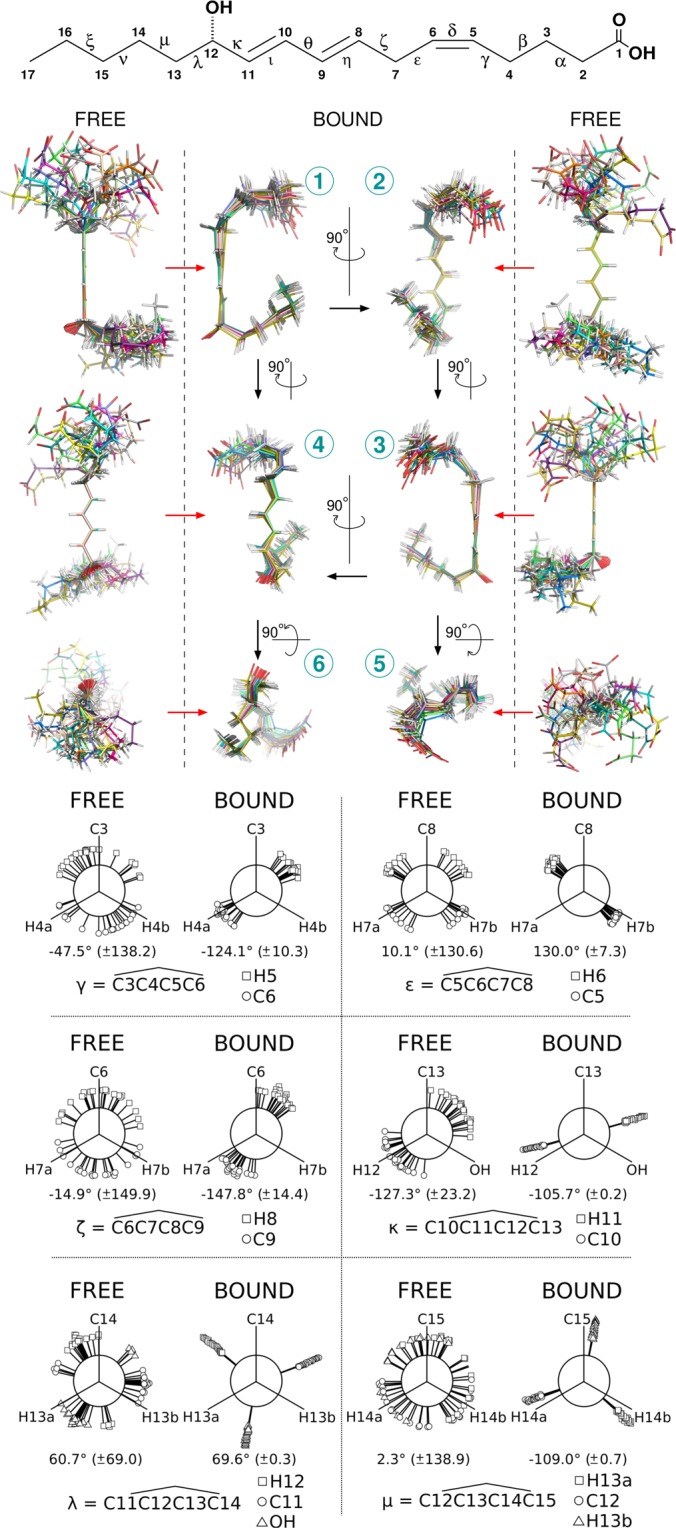
Three-dimensional structure of 12–HHT free in solution or bound to BLT2. (*Top*) Primary chemical structure of 12–HHT. The carbons are numbered from the carboxyl function to the methyl group. Greek letters refer to some dihedral angles displayed at the bottom of the figure. (*Middle*) Six different views of two ensembles of 20 energy-minimized conformers (in *white*, hydrogen atoms; in *red*, oxygen atoms; carbon atoms are assigned a different color for each conformer). The *red* arrows indicate the transition from the *free* to the *bound* state for a same orientation of the diene located at the center of the molecule (carbons C8 to C11). (*Bottom*) Comparison of dihedral angles between the free and the bound states for the set of 20 conformers displayed above.

The NMR data corresponding to only specific interactions were collected at four different Nuclear Overhauser Effect (NOE) mixing times *τ*_*m*_, *i*.*e*. 0.1, 0.2, 0.35 and 0.5 s and integrated for structure calculations (Tables S1–S4). Dipolar interactions between H2, H3 and H17 with the other protons in the ligand were actually enough to obtain a converged set of structures (Tables S1–S4 and Fig. S4). This set of low energy conformers of 12–HHT in its BLT2 bound-state is depicted in Fig. 2 (with associated structural statistics gathered in Table 1). In order to describe any conformational rearrangement of the structure of the ligand upon binding to its receptor, a structure analysis of 12–HHT free in solution was performed (Table S5) and is also indicated in Fig. 2. As expected, in the free state, structure calculation indicates that both aliphatic ends of the molecule are flexible with the coexistence of various rotamers. However, compared to calculations conducted without any experimental restraints, 12–HHT free in solution adopts preferential conformations at the pentyl-end (carbon atoms 13 to 17), precluding any extended conformation along the axis defined by the unsaturated bonds. Compared to the free-state in solution, the ligand describes a well-constrained conformation in the presence of the receptor. In particular, both ends, the carboxyl-end (1-carboxy-pent-4-ene-5-yl chain, carbons C1 to C6) and the pentyl-end (carbon atoms 13 to 17), adopt a unique orientation respectively to the rigid core of the molecule (dihedral angles $\zeta$ and $\kappa$ in Fig. 2).

**Table 1:** Summary of structural constraints and structure statistics for a set of 20 structures of 12–HHT in the presence of BLT2 receptor.

NOE-based distance restraints		NOE-based distance restraints	
Inter-protons *i*, *j* at *τ*_*m*_ = 0.1 s		Inter-protons *i*, *j* at *τ*_*m*_ = 0.2 s	
$|i-j|=1$	3	$|i-j|=1$	3
$|i-j|=2$	2	$|i-j|=2$	3
$|i-j|=2.5$	1	$|i-j|=2.5$	1
$|i-j|=3$	3	$|i-j|=3$	2
$|i-j|=4$	4	$|i-j|=4$	3
$|i-j|=5$	3	$|i-j|=5$	4
$|i-j|=6$	3	$|i-j|=6$	3
$|i-j|=7$	3	$|i-j|=7$	3
$|i-j|=8$	2	$|i-j|=8$	3
$|i-j|=9$	1	$|i-j|=9$	2
$|i-j|=10$	1	$|i-j|=10$	2
$|i-j|=11$	2	$|i-j|=11$	2
$|i-j|=12$	1	$|i-j|=12$	2
$|i-j|=13$	2	$|i-j|=13$	2
$|i-j|=15$	1	$|i-j|=15$	1
Total	32	Total	36
Inter-protons *i*, *j* at *τ*_*m*_ = 0.35 s		Inter-protons *i*, *j* at *τ*_*m*_ = 0.5 s	
$|i-j|=1$	3	$|i-j|=1$	2
$|i-j|=2$	3	$|i-j|=2$	1
$|i-j|=2.5$	1	$|i-j|=2.5$	1
$|i-j|=3$	2	$|i-j|=3$	1
$|i-j|=4$	4	$|i-j|=4$	3
$|i-j|=5$	4	$|i-j|=5$	3
$|i-j|=6$	3	$|i-j|=6$	2
$|i-j|=7$	3	$|i-j|=6.5$	1
$|i-j|=8$	3	$|i-j|=7$	2
$|i-j|=9$	2	$|i-j|=8$	2
$|i-j|=10$	2	$|i-j|=9$	3
$|i-j|=11$	2	$|i-j|=10$	2
$|i-j|=12$	1	$|i-j|=11$	1
$|i-j|=13$	2	$|i-j|=12$	2
$|i-j|=14$	1	$|i-j|=13$	2
$|i-j|=15$	1	$|i-j|=14$	1
		$|i-j|=15$	1
Total	37	Total	30
**Structural statistics**			
Number of NOE violations >0.5 Å		1 ± 0	
Number of NOE violations >0.2 Å		1 ± 0	
Number of NOE violations >0.1 Å		3.4 ± 0.49	
Mean global rms		0.22 ± 0.20 (Å)	
**Deviation from idealized geometry**			
Mean rms bond		4.8 × 10^−3^ ± 5.1 × 10^−5^ (Å)	
Mean rms angle		0.78 ± 5.7 × 10^−3^ (degrees)	
Mean rms improper		2.16 ± 8.2 × 10^−3^ (degrees)	
Mean rms dihedral		0.37 ± 7.0 × 10^−3^ (degrees)	
**Mean energies** (**kcal.mol**^**−1**^)			
E_*bonds*_		1.05 ± 2.2 × 10^−2^	
E_*angles*_		7.53 ± 0.11	
E_*impropers*_		18.12 ± 0.14	
E_*dihedrals*_		0.83 ± 3.2 × 10^−2^	
E_*vdw*_		−9.48 ± 0.16	
E_*total*_		18.06 ± 0.16	

(In the case where the inter-protons distance indicated is not an integer, this means that magnetically not equivalent protons could not be distinguished in the NOESY spectrum. For instance, between H5 and H6, a dipolar interaction with H7 corresponds to an average inter-proton restraints of 2.5).

### Docking model of 12–HHT associated with BLT2 receptor

**Figure 3:**
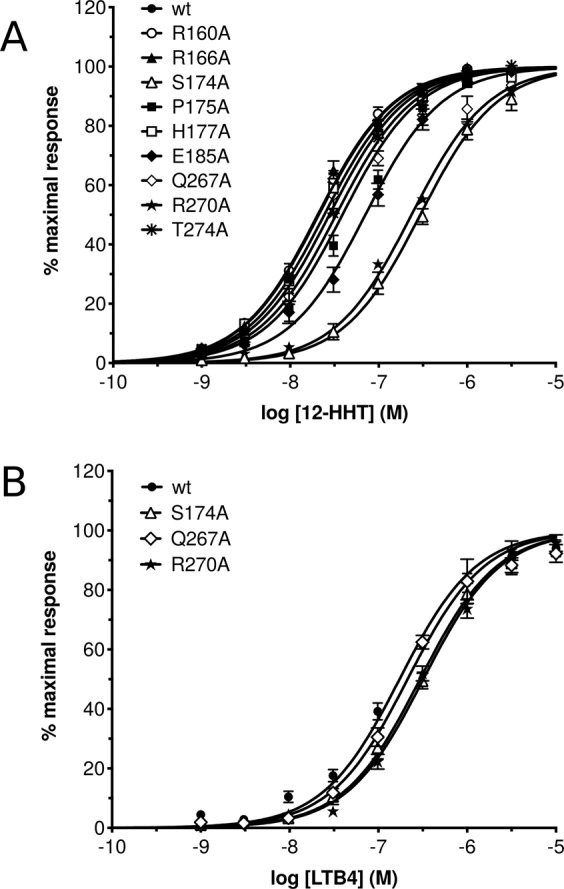
G_*i*_ protein activation catalyzed by the wild-type BLT2 receptor and its mutants in the presence of increasing 12–HHT (**A**) or LTB4 (**B**) concentrations. Data are presented as the mean ± SEM of three experiments.

The set of 20 low energy conformers depicted in Fig. 2 was further integrated in a model of the BLT2 receptor to perform docking simulations using the software HADDOCK^68,69^. The model of the BLT2 receptor using Modeller^59–61^ was based on the active state of the *β*2 adrenergic receptor (*β*2AR) (pdb code 3p0g^4^) in spite a structure of BLT1 associated with an inverse agonist being available in the protein databank (pdb code 5x33^62^). Using BLT1 crystal structure in an inactive state does not allow the ligand 12–HHT to interact with important amino acids in the BLT2 receptor that have been identified by site-directed mutagenesis experiments associated with ligand binding assays (Fig. 3A and Table 2). In particular, residue S174 in the extra-cellular loop 2 (ECL2) BLT1-based BLT2 model is located too far from the top of the ligand orthosteric pocket as the loop is in an open-lid conformation in the inactive state (Fig. S5). To reproduce contacts between the ligand and the receptor based on our binding studies, a conformational homology model was built. In addition, the identity in amino acid sequence between BLT1 and BLT2 is only 45%, which is mostly in the 7TM. Docking simulations were performed for each of the 20 NMR conformers based on two active residues identified by mutagenesis, S174 and R270 (Fig. 3A). Simulations with HADDOCK were started with 12–HHT NMR conformers well away from the orthosteric site of *β*2AR-active-based BLT2 model, *i*.*e*. not partly positioned in the orthosteric site.

**Table 2:** EC50 values inferred from G_*i*_ protein activation catalyzed by the wild-type BLT2 receptor and its mutants in the presence of 12–HHT.

Constructs	LogEC50	EC50
wt	−7.677	2.102 × 10^−8^
R160^4.64^A	−7.689	2.046 × 10^−8^
R166^*ECL*2^A	−7.684	2.071 × 10^−8^
S174^*ECL*2^A	−6.539	2.948 × 10^−7^
P175^*ECL*2^A	−7.410	3.889 × 10^−8^
H177^*ECL*2^A	−7.615	2.427 × 10^−8^
E185^5.42^A	−7.151	7.070 × 10^−8^
Q267^7.32^A	−7.481	3.307 × 10^−8^
R270^7.35^A	−6.629	2.350 × 10^−7^
T274^7.39^A	−7.557	2.771 × 10^−8^

**Figure 4:**
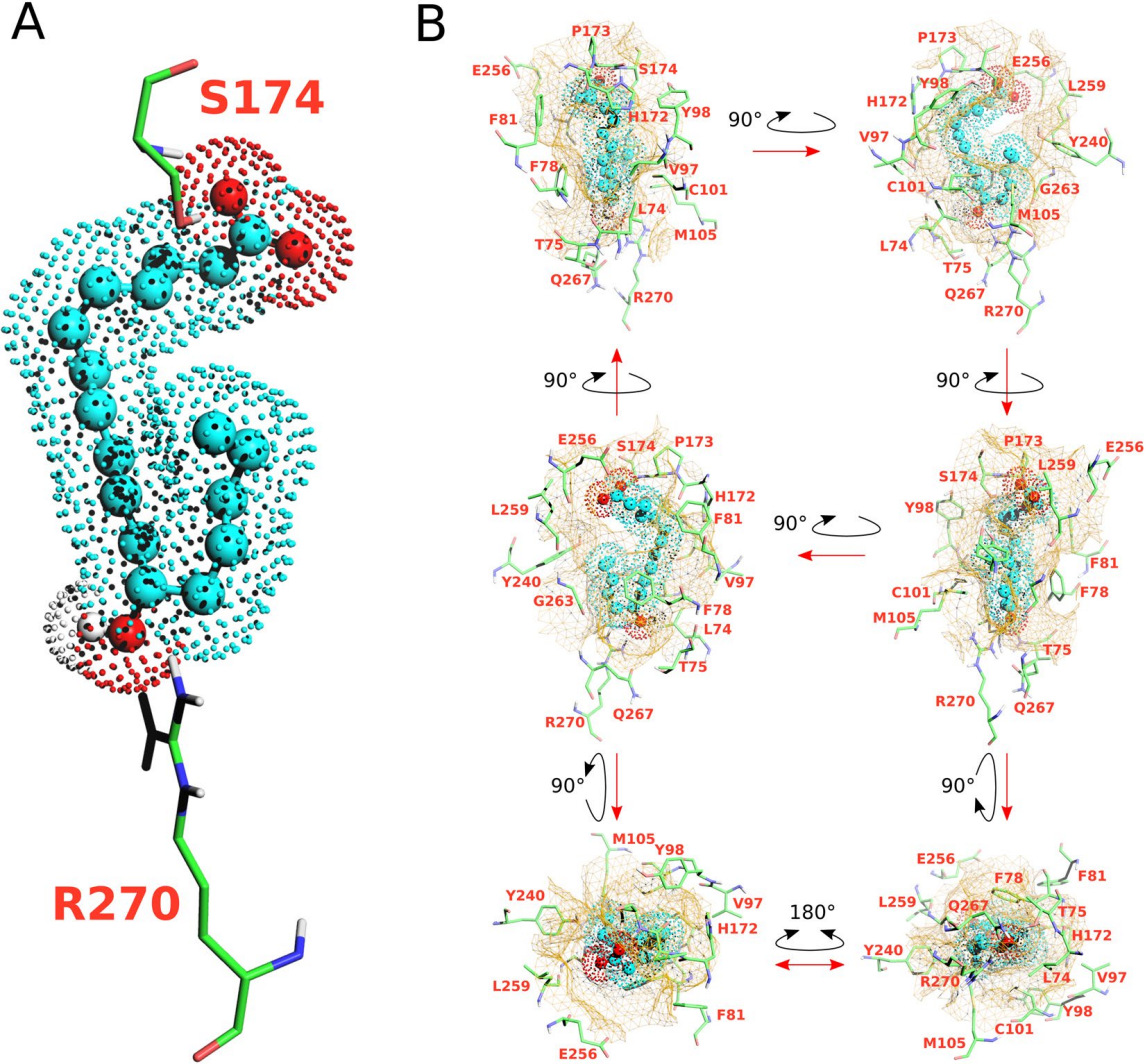
Docking model of the NMR structure of 12–HHT in human BLT2 receptor (see HADDOCK structural statistics in Table S11). (**A**) represents the ligand in spheres and dots (hydrocarbon skeleton in *cyan*, oxygen atoms in *red* and the proton of the hydroxyle group in position 12 in *white*) double-locked at the top and the bottom of the orthosteric pocket by two hydrogen bonds with S174 and R270 residues. (**B**) represents six different views of the ligand in the orthosteric pocket of the receptor. The cavity of the ligand binding pocket is represented with a brown mesh surface at a maximum distance of 5 Å from the ligand. Amino acids delineating the pocket are indicated in *orange*.

All these simulations gave rise to a single cluster or a predominant cluster of structures representing 97 to 100% of the water-refined models generated by HADDOCK. The simulations proposed various possible orientations of the ligand in the orthosteric pocket, but only one orientation depicted in Fig. 4 is compatible with site-directed mutagenesis experiments associated with ligand binding assays (Fig. 3A and Table 2) with a particular focus on the two residues that establish hydrogen bonds with 12–HHT, *i*.*e*. S174^*ECL*2^ and R270^7.35^ (superscripts indicate residue numbering following the Ballesteros-Weinstein nomenclature^70^). Indeed, that orientation shows an excellent agreement with these two single mutations, *i*.*e*. S174^*ECL*2^A and R270^7.35^A, with EC50 values shifted from 21 nM (wild-type) to 295 nM for S174^*ECL*2^A and 235 nM for R270^7.35^A. In that position, S174^*ECL*2^ establishes hydrogen bonds with the carboxylate group of 12-HHT and R270^7.35^ interacts with the hydroxyl moiety of the ligand through hydrogen bonds as well (Fig. 4). A similar position of the ligand that came out from the simulations involves an additional hydrogen bond between the hydroxyl group of the ligand and Q267^7.32^, but as no significant change in ligand binding could be observed by introducing the mutation Q267^7.32^A (Fig. 3A and Table 2), that orientation was discarded.

The model of the interaction of 12–HHT NMR structure with a model of BLT2 based on an active conformation of *β*2AR displays interactions with five secondary elements in the receptor: 4 helices (II, III, VI and VII), which delineate the contours of the orthosteric pocket, and one extra-cellular loop (ECL2) which plays a role of lid above the ligand pocket (Fig. 4). In addition to the two amino acids that establish hydrogen bonds with the ligand (S174^*ECL*2^ and R270^7.35^), 10 other amino acids located at a distance ≤4 *Å* from the ligand (20 amino acids located at a distance ≤5 *Å* depicted in Fig. 4) show various weak interactions, including CH-to-*π*, CH-to-O, NH-to-*π*, S-to-CH or N-to-CH proximities. The model of interaction depicted in Fig. 4 is also in accordance with some other neutral mutations that have been tested: first, residues that are important for LTB4 binding to BLT1. R160^4.64^, which is highly conserved in both BLT1 and BLT2 receptors (Fig. S6), has been identified to be crucial for LTB4 binding on BLT1 (residue R156 in BLT1) by potentially making a direct hydrogen bond with the carboxylate head group^71^. Mutation of this residue to alanine results in a complete loss of LTB4 binding^71^. Accordingly to our model, in which R160^4.64^ is located very far from 12–HHT (Fig. S7), R160^4.64^A mutant has no effect on 12–HHT binding (Fig. 3A and Table 2). In the same way, the E185A mutation did not significantly affect 12–HHT binding (Fig. 3A) whereas mutating this residue had a noticeable impact on LTB4 binding onto BLT1^71^. Second, some neutral mutations have been conducted. Just beside S174^*ECL*2^ in ECL2, but not establishing any interaction with the ligand, P175^*ECL*2^ and H177^*ECL*2^, which mutations to alanine do not display a significant impact on ligand binding compared to the wild-type receptor. Another residue in ECL2, which could possibly interact with the carboxyl function of the ligand, R166^*ECL*2^, and an additional neutral mutation close to R270^7.35^, T274^7.39^A, do not impact receptor ligand properties (Fig. 3A and Table 2) in accordance with our model.

### Comparison of 12–HHT and LTB4 structures in their BLT2-bound states

**Figure 5:**
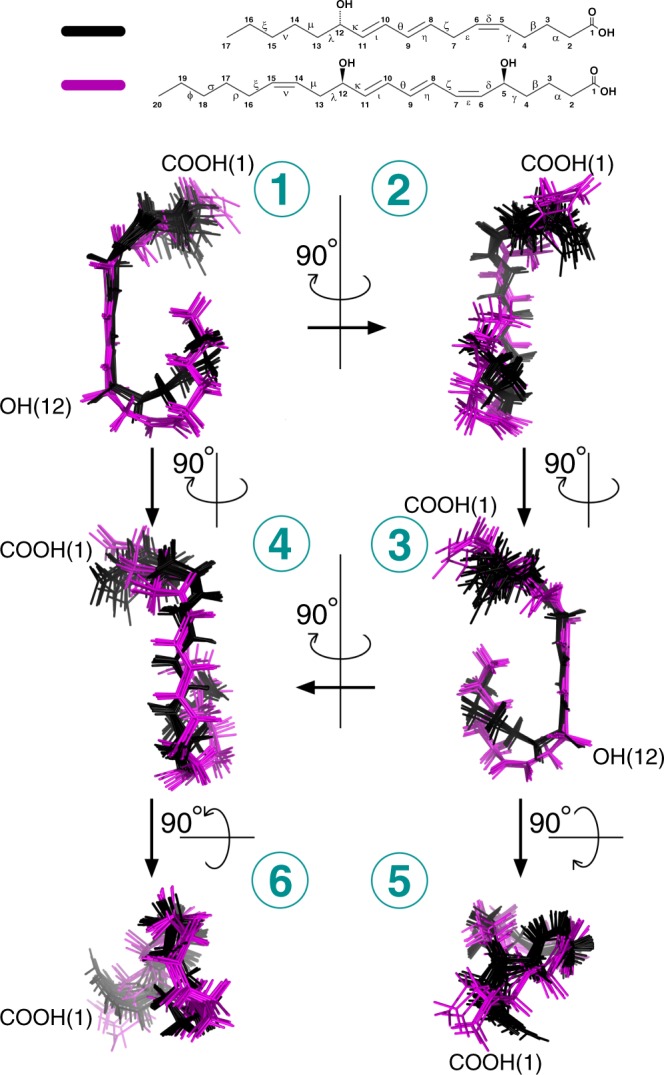
Comparison of 12–HHT and LTB4 3D NMR structures associated with human BLT2. Six different views of superimposed ensembles of 20 energy-minimized conformers of 12–HHT (in *black*, from Fig. 2), and the 7 lowest energy conformers of LTB4 (in *purple*, from Fig. S9). On *Top* are represented the chemical structures of the ligands.

We present also in this study a new set of converged structures of LTB4 associated with BLT2 in order to compare the bound structures of 12–HHT and LTB4 obtained under identical conditions and procedures. Compared to the first calculation published in 2010^18^, NMR data were collected at 700 MHz with a receptor associated with perDAPol^50^ instead of DAPol and by using a softer methodology to remove the ^1^H signal of H_2_O to not affect signal intensities from the ligand (see Material and Methods). As observed with 12–HHT, in the absence of the receptor, both ends of the ligand are not structured, based on the observation of intra-ligand ^1^H–to–^1^H dipolar interactions (Fig. S8). Calculation based on NMR data collected in the presence of BLT2 gives rise to a folded structure (Fig. S9 and Table S10) similar to the previous published structure^18^, but with an orientation of the carboxyl-end (carbons 1 to 5) more loosely defined if we take into account an ensemble of 15 or 20 NMR structures (Fig. S9 and see dihedral $\zeta$ in Figure S10). If we try to coincide the lowest energy conformers of LTB4 with the 12–HHT structure ensemble by superimposing the most rigid part of the hydrocarbon skeletons, *i*.*e*. carbons 7 to 12, we find that, globally, the fold of LTB4 is close to the 12–HHT structure in the presence of the same receptor (Fig. 5): the orientation of the carboxyl-end is similar, but not identical, the hydroxyl group in position 12 points towards the same direction despite an opposite chirality of the asymmetric carbon, and the methyl end for both ligands are quite close despite the LTB4 chain containing three more carbons. However, the two chains from carbon 12 –bearing the hydroxyl group– to the methyl end display different orientations (see views *1* and *3* in Fig. 5). This region of these ligands is supposed to be located at the bottom of the pocket of the receptor, based on the grafting of fluorescent probes at the carboxyl-end on the LTB4 for instance that does not affect the binding properties to BLT2^72^.

Attempts to get a model of LTB4 associated with BLT2 failed because we could not identify clear mutants that impact significantly on the binding of LTB4 onto BLT2, and this prevented us from getting a reasonable model of the ligand:receptor complex. Furthermore, in contrast to 12–HHT, LTB4 is a very low-affinity ligand for BLT2, and this certainly contributes to the fact that we could not get any satisfying model for this ligand.

## Discussion

Historically BLT2 was designated as the low-affinity LTB4 receptor, in contrast to BLT1, with *in cellulo* K_*d*_ of ~20 nM compared with ~1 nM for BLT1^30^. More recently, strong evidences led to the discovery of BLT2 endogenous agonist, 12–HHT^33,38,41^, a non-eicosanoid fatty acid compound which essentially comes from the conversion of prostaglandin H2 to thromboxane A2. *In cellulo* measurements indicate a higher affinity of 12–HHT for BLT2 compared to LTB4, by about one order of magnitude^38^. This was also observed by *in vitro* binding measurements of LTB4 and 12–HHT onto a purified BLT2 receptor associated with amphipols in solution, with K_*d*_ of ~200 and ~60 nM, respectively^19^. To be noted, the affinity of the isolated receptor for its agonists is lower than that measured in cell systems. However, high affinity can be recovered by associating the isolated receptor with its cognate G proteins^51^. Hence, the structures obtained here with the isolated BLT2 are likely signatures of the low-affinity, uncoupled state of the receptor. A qualitative comparison of NMR NOESY spectra clearly indicate an organization of both ends of 12–HHT in the presence of BLT2 (Figs. S2 and S3). Structure calculation confirmed that observation and led to a single set of converged structures (Fig. 2). The model proposed herein describes a ligand that is double-locked in the receptor by two hydrogen bonds which are in accordance with single-directed mutagenesis associated with ligand binding experiments (Fig. 3A): one NH$\cdot \cdot \cdot$O hydrogen bond between the OH moiety of the ligand and R270^7.35^ at the bottom of the orthosteric pocket, and a stronger OH$\cdot \cdot \cdot$O hydrogen bond involving the COOH group of 12–HHT with S274^*ECL*2^, *i*.*e*. on the opposite side of the ligand pocket (Fig. 4). These two residues are highly conserved in BLT receptors (Fig. S6), but interestingly, while BLT1 is almost activated by the LTB4 only, and not by 12–HHT, mutation of these two residues does not affect the binding of LTB4 onto BLT2 (Fig. 3B and Table 3). In complement to measurements at equilibrium, *in vitro* off-rate constant measurements led to a bound time 3.6 times longer for 12–HHT than LTB4^19^. This also tends to suggest additional short range interactions for 12–HHT compared with LTB4. A tentative superimposition of 12–HHT and LTB4 in their BLT2-bound states described a similar fold, especially if we take into account the 7 lowest energy structures obtained in the converged ensemble of structures of LTB4 (Fig. 5 and Table S10). However, several main features distinguish 12–HHT and LTB4 than could explain these different binding properties: a shorter hydrocarbon chain for 12–HHT, with a double bond less, the absence of a hydroxyl group on position 5, and an opposite chirality for the asymmetric carbone 12 (see top of Fig. 5). In addition, superimposing the rigid core of these two ligands indicates noticeable differences, especially from the asymmetric carbon 12 to the methyl end (Fig. 5), a region which should interact with the bottom of the orthosteric pocket.

**Table 3:** EC50 values inferred from G_*i*_ protein activation catalyzed by the wild-type BLT2 receptor and its mutants in the presence of LTB4.

Constructs	LogEC50	EC50
wt	–7.677	2.102 × 10^−8^
R160^4.64^A	–7.689	2.046 × 10^−8^
R166^*ECL*2^A	–7.684	2.071 × 10^−8^
S174^*ECL*2^A	–6.539	2.948 × 10^−7^
P175^*ECL*2^A	–7.410	3.889 × 10^−8^
H177^*ECL*2^A	–7.615	2.427 × 10^−8^
E185^5.42^A	–7.151	7.070 × 10^−8^
Q267^7.32^A	–7.481	3.307 × 10^−8^
R270^7.35^A	–6.629	2.350 × 10^−7^
T274^7.39^A	–7.557	2.771 × 10^−8^

Structures have revealed that a high percentage of identity between sub-families of class A GPCRs can be observed for amino acids sculpting the orthosteric binding pocket in contrast with the extracellular domains and membrane interface, which comprise the N-terminus end and three extracellular loops and the top of the TMs. These regions display a higher diversity in both sequence and length^73^. Experimental data indicate that ECLs are intimately implicated in GPCR activation^74^. Compilation of that information suggests a role of these extracellular regions in GPCR signaling, including ligand binding and selectivity^75^ in addition to ligand efficacy^76^, allosteric modulations, *e*.*g*.^77^ and constitutive activation^78^. Our model based on the crystallographic active state of the *β*2AR highlights the importance of ECL2 as a lock above the orthosteric site which one residue, S174, display the strongest interaction above all residues that interact with the ligand. Two additional residues, *i*.*e*. H172 and P173, associated with S174 define a hood above the ligand that probably contribute to improve the residence time of the ligand to promote the binding of an intracellular partner as equilibrium binding properties may not totally govern the activation of GPCRs. In other words, non-equilibrium kinetics of the ligand binding event may also play an important role^79^.

The method proposed in this study deserves to be improved as some imperfections could introduce biases in both the structure calculation and also in the model. First of all, the definition of parameter and topology files for organic compounds is not so trivial despite the development of very efficient and convenient programs like PRODRG^53^ and XPLO2D^54^ that have been used in the present study. Structure calculations were based on the program ARIA^55^ associated with CNS^56^ which contains a full relaxation matrix treatment of NOE data to take into account indirect ^1^H–^1^H cross-relaxation pathways^57,58^ but does not take into account the contribution of the chemical exchange of the ligand from the receptor in the calculation. It would be interesting to include a matrix of exchange to properly gauge the impact of the *k*_*off*_ –or conversely the residence time– of the ligand in the structure calculation. In the present study, the receptor is perdeuterated (98%) in order to limit the spin diffusion into the ligand only, *i*.*e*. not relayed by protons of the protein, but the remaining 2% of protons in the receptor may slightly impact also the intra-ligand dipolar restraints observed by NMR. We also tried to be as cautious as possible to use specific intra-dipolar interactions only in the structure calculations, but this does not exclude some imperfections in the approach. For all these reasons, we cannot exclude that both flexible ends of 12–HHT (carbons 1 to 4 and 13 to 17) may display slightly different orientations compared to the set of structures described herein. It should be noted that in the recent published structure of the protaglandin E2 bound to EP3 receptor^80^, the ligand displays also a non-extended conformation in accordance with our results (Fig. S11). In addition, docking simulations with the set of conformers of free 12–HHT in solution (see Fig. 2) could not reproduce the contacts observed between 12–HHT BLT2-bound structures and S174 and R270 (Fig. S12). However, to help us to improve the method, NOE peak volumes for both 12–HHT and LTB4 are available to the community in Tables S1–S4 and S6–S9, respectively. Ideally, the experimental determination of a high-resolution structure of BLT2 receptor associated with 12–HHT would greatly help to adjust the approach detailed here. Other biophysical methods like NMR chemical shift perturbation experiments with a specifically isotope-labeled BLT2 receptor, crosslinking and/or hydrogen/deuterium exchange associated with mass spectrometry, and also molecular dynamics simulations could help to improve the model proposed in the present study by determining some contact between the ligand and some amino acids of the receptor. These methods have been used in the GPCR field to delineate ligand:receptor contacts^81–83^ and probe the changes in receptor conformation induced by the interaction with the ligands^84^. Overall, our data bring a first description of 12–HHT in its receptor-bound state. This demonstrates the interest of a NMR-based approach to provide a description of the structure and dynamics of natural ligands bound to unmodified receptors at physiological temperatures, in complement to X-ray crystallography and cryoEM methods.

## Supplementary information

Supplementary Information.
