# Supplementary material for: Structure of the agonist 12–HHT in its BLT2 receptor-bound state
Source: Sci Rep. 2020 Feb 14;10:2630. doi: 10.1038/s41598-020-59571-6 (PMC7021728; doi:10.1038/s41598-020-59571-6)
Supplement: Supplementary file 1 — Supplementary Information. [file 41598_2020_59571_MOESM1_ESM.pdf]

Supplementary Information

Structure of the agonist 12–HHT in its BLT2 receptor-bound state

Fabrice Giusti,[1,2] Marina Casiraghi,[1,3] Elodie Point,[1] Marjorie Damian,[4] Jutta Rieger,[5] Christel Le Bon,[1] Alexandre Pozza,[1] Karine Moncoq,[1] Jean-Louis Banères,[4] Laurent J. Catoire[1,*]

[1]Laboratoire de Biologie Physico-Chimique des Protéines Membranaires, UMR 7099, CNRS/ Université de Paris, Institut de Biologie Physico–Chimique (FRC 550), 13 rue Pierre et Marie Curie, F–75005 Paris, France; [2]Present address: Institut de Chimie Séparative de Marcoule, ICSM UMR 5257, Site de Marcoule, Bâtiment 426, BP 17171, F-30207 Bagnols sur Cèze Cedex, France; [3]Present address: Department of Molecular and Cellular Physiology, Stanford University School of Medicine, 279 Campus Drive, 94305 Stanford California, USA; [4]Institut des Biomolécules Max Mousseron (IBMM), UMR 5247 CNRS, Université Montpellier, ENSCM, 15 av. Charles Flahault, 34093 Montpellier, France; [5]Institut Parisien de Chimie Moléculaire, Sorbonne Université, CNRS, UMR 8232, Equipe Chimie des Polymères, 4 place Jussieu, 75252, Paris Cedex 05, France

– Tables S1 to S11

– Figures S1 to S12

– References

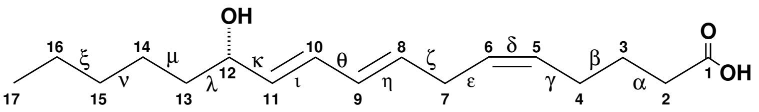

$\tau_m = 0.1$ s

spins	H2	H3	H4	H5	H6	H7	H8	H9	H10	H11	H12	H13	H14+H15+H16	H17
H2		41817		6752	5246	7429		1552	526	∼0	∼0			1802
H3	37108		34516	11562	6854	8510					∼0			
H4		35376		16619	12179	30397	4726	4579	2495	2751	∼0			2640
H5+H6	8701	15465	25500			25066	6049	7002	3076				4348	1921
H7	7987	7360	34349	14483	25881		14915	17567	8088	5442	1365			1335
H8	1705	1233	4320			15094		14158	28359	8106	4952	2549	4443	983
H9+H10	2295	1789	5655	6534	8183	22927	36360			37908	25397	11642	18919	2836
H11	∼0	∼0				8795	7963	30490	15070		8967	17155	16832	2130
H12	∼0	∼0	∼0	774	1152	670	4429	7576	17840	8878		21920	29996	2846
H13	∼0		∼0	∼0	∼0	∼0					28267		130530	
H14 H15 H16	4267		4560	2230	2000	4283	2879	7513	11521	12587	32693	95330		78777
H17	1335		1481	1577	704	2285	1426	1469	1003	1538	2285	11233	73101	

TABLE S1. Related to Figure 1, Figure 2 and Table 1. ^{1}H–^{1}H NOE peak volumes of 12–HHT measured in the presence of human BLT2 receptor in a two-dimensional NOESY experiment (mixing time τ_m = **0.1 s**). The color code indicate the proportion of non-specific binding estimated with the ligand in the presence of perDAPol only in the same experimental conditions: boxes colored in dark green ■, light green ■, yellow ■ and red ■ indicate a potential proportion of non-specific binding <10%, [10%, 20%[, [20%,50%[and above 50%, respectively. Boxes colored in gray ■ designed dipolar interaction that could not be measured at this mixing time.

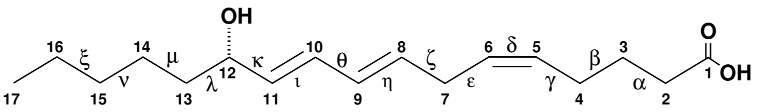

$\tau_m = 0.2$ s

spins	H2	H3	H4	H5	H6	H7	H8	H9	H10	H11	H12	H13	H14+H15+H16	H17
H2		52601		9937	9105	14534	2735	3442	2905	2877	854		11845	3596
H3	49508		43989											
H4		44820		23638	18334	40157	6664	8416	4776	4154	2670		15802	4980
H5+H6	14415	25128	36961			37557	8870	12699	5805		1964		9261	4356
H7	13572	14824	44466	21332	24553		17207	21267	13022	9369	3871	4873	13339	4643
H8	3035	3789	7552	5641	7141	18772		15832	26206	7950	9843	6268	12879	3129
H9+H10	4660	7384	12297	10928	11609	29417	37096			39035	27937	16694	36868	6685
H11						10595	9799	27232	15794		11614	20267	30442	3878
H12	588		796	1121	1172	2993	7334	9513	17195	10140		29259	44236	5769
H13													156730	
H14 H15 H16	9792		12496	7377	8262	11991	10338	16385	20116	23456	46335	115610		125150
H17	4407	7235	4428	2994	1920	3284	1717	2818	2762	3236	5467	19338	107480	

TABLE S2. Related to Figure 1, Figure 2 and Table 1. ^{1}H–^{1}H NOE peak volumes of 12–HHT measured in the presence of human BLT2 receptor in a two-dimensional NOESY experiment (mixing time τ_m = **0.2 s**). The color code indicate the proportion of non-specific binding estimated with the ligand in the presence of perDAPol only in the same experimental conditions: boxes colored in dark green, light green, yellow and red indicate a potential proportion of non-specific binding <10%, [10%, 20%[, [20%,50%[and above 50%, respectively. Boxes colored in gray designed dipolar interaction that could not be measured at this mixing time.

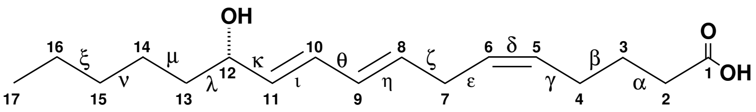

$\tau_m = 0.35$ s

spins	H2	H3	H4	H5	H6	H7	H8	H9	H10	H11	H12	H13	H14+H15+H16	H17
H2		49233	48853	12919	10750	14523	4102	5005	3123	3142	1905		16888	6923
H3														
H4	50308	47014		24785	20715	35386	9364	9740	7104	7093	3138		21041	7684
H5+H6	18546	30105	28257			33948	8822	11593	7407		4065		17795	6611
H7	15822	19134	36786	20529	22884		14967	18100	11788	10785	4995		49134	7251
H8	3969	5223	8725	5829	6935	15748		11744	16249	7412	9041	8523	17897	4353
H9+H10	7171	9418	14024	11523	12117	25353	24479			25271	20422	19937	48236	10435
H11						11478	7139	18168	11627		10399	16844	35398	7476
H12	1295		2722	2430	2658	4447	7810	7398	12232	8183		18120	42708	8310
H13														
H14 H15 H16	13344		17260	11765	12493	17286	17915	25074	28095	30405	43353	101780		142100
H17	5451	11298	6846	3313	3311	5627	2844	4333	5292	5058	8424	22302	123490	

Table S3. Related to Figure 1, Figure 2 and Table 1. ^{1}H–^{1}H NOE peak volumes of 12–HHT measured in the presence of human BLT2 receptor in a two-dimensional NOESY experiment (mixing time τ_m = **0.35 s**). The color code indicate the proportion of non-specific binding estimated with the ligand in the presence of perDAPol only in the same experimental conditions: boxes colored in dark green, light green, yellow and red indicate a potential proportion of non-specific binding <10%, [10%, 20%[, [20%,50%[and above 50%, respectively. Boxes colored in gray designed dipolar interaction that could not be measured at this mixing time.

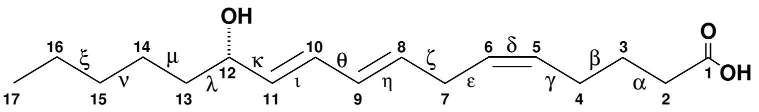

$\tau_m = 0.5$ s

spins	H2	H3	H4	H5	H6	H7	H8	H9	H10	H11	H12	H13	H14+H15+H16	H17
H2		49080		15470	12045	16966	5851	5281	5142	5127	2952		20893	8835
H3														
H4		38630		21019	21040	32681	8646	9659	7524	7784	6057		25461	11907
H5+H6	23627	31110	39079			33325	9846	11069	8839		5423		29129	10846
H7	17393	18651	32780	19545	20058		13074	14682	12904	11266	6261		26871	10697
H8	5628	6796	9873	6393	6567	13551		9570	12345	7682	7751	8704	23776	6963
H9+H10	10308	11265	16105	12575	12512	22537	19732			22104	16588	22818	56376	15788
H11						10815	7487	12929	10136		8985	15154	39081	9954
H12	2311	3623	4779	2389	3203	4870	6498	7235	8569	7245		15219	46042	12935
H13														
H14 H15 H16	19095	31353	22040	16280	16961	23773	21876	27712	32033	35467	45110	117825		166000
H17	7587	12797	8919	5361	6560	7638	5447	7085	8715	7862	9465	31477	149060	

TABLE S4. Related to Figure 1, Figure 2 and Table 1. ^{1}H–^{1}H NOE peak volumes of 12–HHT measured in the presence of human BLT2 receptor in a two-dimensional NOESY experiment (mixing time τ_m = **0.5 s**). The color code indicate the proportion of non-specific binding estimated with the ligand in the presence of perDAPol only in the same experimental conditions: boxes colored in dark green, light green, yellow and red indicate a potential proportion of non-specific binding <10%, [10%, 20%[, [20%,50%[and above 50%, respectively. Boxes colored in gray designed dipolar interaction that could not be measured at this mixing time.

**NOE-based distance restraints**	
Inter-protons i,j	
$\mid i-j \mid = 1$	5
$\mid i-j \mid = 2$	5
$\mid i-j \mid = 3$	2
Total	12
**Structural statistics**	
Number of NOE violations > 0.1 Å	0
Mean global rms	1.53 ± 0.52 (Å)
**Deviation from idealized geometry**	
Mean rms bond	$4.5\times10^{-3} \pm 10^{-4}$ (Å)
Mean rms angle	$0.76 \pm 8.8\times10^{-3}$ (degrees)
Mean rms improper	$2.12 \pm 8.8\times10^{-3}$ (degrees)
Mean rms dihedral	$0.26 \pm 8.6\times10^{-3}$ (degrees)
**Mean energies (kcal.mol^{-1})**	
E_{bonds}	$0.89 \pm 4.30\times10^{-2}$
E_{angles}	7.12 ± 0.16
$E_{impropers}$	17.39 ± 0.14
$E_{dihedrals}$	$0.43 \pm 2.8\times10^{-2}$
E_{vdw}	-8.28 ± 0.40
E_{total}	17.51 ± 0.40

Table S5. Related to Figure 2. Summary of structural constraints and structure statistics for a set of 20 structures of 12–HHT free in solution.

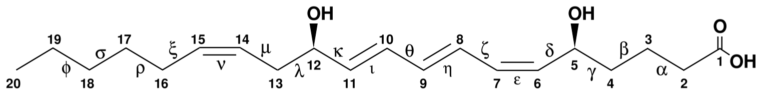

spins	H2	H3	H4	H5	H6+H14	H7	H8	H9	H10	H11	H12	H13	H15	H16	H17+H18+H19	H20
H2		25522	17740			~0	~0	~0		~0	~0		~0			
H3	37668				7474	1965	2961	~0	719	147	~0		~0			
H4					4456	654	3468	~0	~0	~0	~0		~0			
H5	1526	5378	7024		3823	1676	14757	868	3105	~0	566	~0	~0	~0		
H6+H14	1693	3817	4708			16415	2192	3336	2741	4588	5009	10249		11990	4696-6424	
H7	73		341		22714		2445	14886	5776	3755	265	~0		~0		
H8	229	2747	3482		2317	3648		7509	11440	965	1330	~0	~0	~0		
H9	~0	~0	~0		10982	27553	14407			22149	12816	3458	~0	~0		
H10	395	389	~0										~0	~0		
H11	119	~0	~0			3606	254	17480	8767		7454	4447		1938	1298 (H17only)	
H12	~0				4416	~0	759	3336	8245	5668		9457	~0	2442		
H13					16511	~0	~0	1674	3040	7235	14105		~0	32714		
H15	~0	~0	~0				~0	~0				~0				
H16					15435	~0	~0	~0	~0	456	1518	21404	965			
H17+H18+H19		127410			10893	~0	~0	737	727	203	1728		~0			119680
H20	1902				~0	~0	~0	382	~0	~0	481		~0	3743	109240	

TABLE S6. Related to Figure 5. ^{1}H–^{1}H NOE peak volumes of LTB4 measured in the presence of human BLT2 receptor in a two-dimensional NOESY experiment (mixing time τ_m = **0.05 s**). The color code indicate the proportion of non-specific binding estimated with the ligand in the presence of perDAPol only in the same conditions experimental conditions: only boxes colored in orange potentially display a non-negligeable contribution of non-specific binding. Boxes colored in gray designed dipolar interaction that could not be measured at this mixing time.

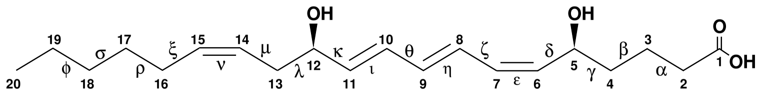

spins	H2	H3	H4	H5	H6+H14	H7	H8	H9	H10	H11	H12	H13	H15	H16	H17+H18+H19	H20
H2		25498	27133			682	1437						~0			
H3	46656				11907	2784	5395	1717	2628	~0			~0			
H4					8596	2318	3429	1074	1298	~0			~0			
H5	1774	9805	11188		6663	2549	13906	2920	5479		1343		~0			
H6+H14	4361	7999	10832			20053	4661	9598	6316	8146	6366	18587		24505	23167	2887
H7	545	3237	3907		26646		4682	15686	7057	6677	1314	489	~0	~0		
H8	2081	5790	6989		6211	4616		8319	11892	2608	3860	782	~0	~0	1077-2351	
H9		1749	870				6143				18868	3331	511	1168		
H10	1300	1515	1840				12086				****	4033	~0	1265		
H11		790			10041	6667	2298	19606	13894		10172	8863	~0	5211		
H12	~0	~0	~0		9603	890	2065	6636	10796	8126		13699	~0	5072		
H13					26992	~0	618	3545	5681	10442	17612		1129	51134		
H15	~0	~0	~0			~0	~0					~0				
H16					25393	~0	~0	1494	1572	2841	5493	33192	2061			11141
H17+H18+H19					27102	532	367	684	475	1069	3539	12757		76515		187010
H20					2902	~0	~0	~0	~0	~0	508		~0	8026		

Table S7. Related to Figure 5. ^{1}H–^{1}H NOE peak volumes of LTB4 measured in the presence of human BLT2 receptor in a two-dimensional NOESY experiment (mixing time τ_m = **0.1 s**). The color code indicate the proportion of non-specific binding estimated with the ligand in the presence of perDAPol only in the same conditions experimental conditions: only boxes colored in orange potentially display a non-negligeable contribution of non-specific binding. Boxes colored in gray designed dipolar interaction that could not be measured at this mixing time.

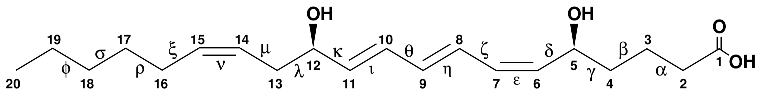

spins	H2	H3	H4	H5	H6+H14	H7	H8	H9	H10	H11	H12	H13	H15	H16	H17+H18+H19	H20
H2		42624	38209				2508						∼0			2543
H3					14883	5014	6544	4426	5062	2150	3287		∼0		194340	
H4					11520	3236	4517	2860	2295	1891	1647		∼0			
H5	3327	10732	12217		6259	3497	6819	3009	4803	1650	1260	1299	∼0	528		
H6+H14	6045	9226	10566			12699	3920	11118	9254	9950	10009	20819		28537	43160	5626
H7	1889	4739	5350		19762		4143	9327	4557	5916	2013	1670	429	1124	3044	659
H8	2022	6173	6702		7004	4328		5302	7006	3085	4544	1737	∼0	1242	2810	1738
H9	1470	2736	3150		17379	11394	5400			15608	6515	4346	827	3070	4864	1063
H10	1768	3344			10103	5939	7519			12037	9650	6089	∼0	3319	2551-3657	1222
H11	956	1434	2493		12766	5540	2133	11889	11833		8264	9521	∼0	5003	4717-5310	1534
H12	947				12001	1560	4803	6060	7757	8132		11899	∼0	7241		
H13					35132	2402	1350	6078	7132	9742	18956			46830		3099
H15	∼0	∼0	∼0								982					
H16					35316	697	432	2262	2387	4569	7862	39963	3725		129810	13884
H17+H18+H19	43084	168440			41029	1560	1164	4107	2872	4517	6597	26885	2806	107560		317320
H20		30459			5992	∼0	∼0	578	881	755			∼0	13836	309430	

TABLE S8. Related to Figure 5. ^{1}H–^{1}H NOE peak volumes of LTB4 measured in the presence of human BLT2 receptor in a two-dimensional NOESY experiment (mixing time τ_m = **0.2 s**). The color code indicate the proportion of non-specific binding estimated with the ligand in the presence of perDAPol only in the same conditions experimental conditions: only boxes colored in orange potentially display a non-negligeable contribution of non-specific binding. Boxes colored in gray designed dipolar interaction that could not be measured at this mixing time.

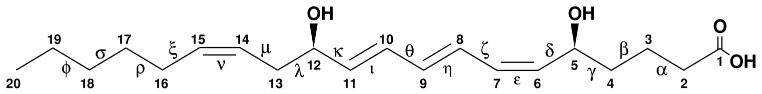

spins	H2	H3	H4	H5	H6+H14	H7	H8	H9	H10	H11	H12	H13	H15	H16	H17+H18+H19	H20
H2		30976	21525		10297	2230	2514	2611	3702	1603	1776		665			
H3	37819				15161	4583	4198	6334	7091	4112	4823		216		207290	
H4					10047	4031	2306		2808	2364	2710		555			
H5	3083	5889	7366		5705	1472	1183	1308	1655	1113	1545	1360		1536	8562	1392
H6+H14	8994	9354	12853			6028	5801	8930	8700	9762	8422	20735		28501	61534	14364
H7	2426	4754	6500		9456		2115	3476	3860	2984	2542	2142	∼0	2990	8455	2293
H8	2352	4267	5655		8181	1832		2710	2127	1929	2870	2572	∼0	2577	6926	3315
H9	3648	3690	4718		10313	3601	1952			4913	8181	4805	∼0	4509	13271	3329
H10	2662	3874	4385		10769	2326	2219			10045		4850	∼0	4601	12487	2300
H11	1437	3272	3415		14524	3490	2018	6253	8128		3547	5541	∼0	6427	15448	3200
H12	1403	2423	1316		10182	1633	1091	3497	4227	3312		5741		6320	15442	4044
H13					32947	3282	1475	5945	6411	8748	7261		3096	31069		10570
H15						∼0	∼0	∼0	∼0	∼0	∼0					
H16					31764	1310	2018	3196	5361	5097	5311	22961	7257		176740	26956
H17+H18+H19	71029	152420			63863	4320	3337	9541	8277	10762	12870	37991		148860		394430
H20	2144	28340			11887	897	1185	2172	1480	1582	2858	7829		19636	335470	

TABLE S9. Related to Figure 5. ^{1}H–^{1}H NOE peak volumes of LTB4 measured in the presence of human BLT2 receptor in a two-dimensional NOESY experiment (mixing time τ_m = **0.5 s**). The color code indicate the proportion of non-specific binding estimated with the ligand in the presence of perDAPol only in the same conditions experimental conditions: only boxes colored in orange potentially display a non-negligeable contribution of non-specific binding. Boxes colored in gray designed dipolar interaction that could not be measured at this mixing time.

**structure number**	**total energy (kcal/mol)**		
**structure_1.pdb**	14.5185	**structure_11.pdb**	16.2504
**structure_2.pdb**	14.7107	**structure_12.pdb**	16.2593
**structure_3.pdb**	14.7849	**structure_13.pdb**	16.4290
**structure_4.pdb**	15.2770	**structure_14.pdb**	16.6447
**structure_5.pdb**	15.5567	**structure_15.pdb**	16.7013
**structure_6.pdb**	15.6157	**structure_16.pdb**	18.3893
**structure_7.pdb**	15.6742	**structure_17.pdb**	19.2519
**structure_8.pdb**	15.6764	**structure_18.pdb**	19.3336
**structure_9.pdb**	15.6826	**structure_19.pdb**	19.7166
**structure_10.pdb**	15.9855	**structure_20.pdb**	19.9861

**NOE-based distance restraints** Inter-protons i,j at $\tau_m = 0.05$ s		**NOE-based distance restraints** Inter-protons i,j at $\tau_m = 0.1$ s	
$\mid i - j \mid = 1$	3	$\mid i - j \mid = 1$	2
$\mid i - j \mid = 2$	1	$\mid i - j \mid = 2$	1
$\mid i - j \mid = 3$	3	$\mid i - j \mid = 3$	2
$\mid i - j \mid = 4$	4	$\mid i - j \mid = 4$	3
$\mid i - j \mid = 5$	4	$\mid i - j \mid = 5$	4
$\mid i - j \mid = 6$	7	$\mid i - j \mid = 6$	9
$\mid i - j \mid = 7$	3	$\mid i - j \mid = 7$	4
$\mid i - j \mid = 8$	4	$\mid i - j \mid = 8$	6
$\mid i - j \mid = 8.5$	1	$\mid i - j \mid = 9$	2
$\mid i - j \mid = 9$	1	$\mid i - j \mid = 10$	1
$\mid i - j \mid = 10.5$	1	$\mid i - j \mid = 10.5$	1
		$\mid i - j \mid = 11$	1
Total	32	Total	36

Inter-protons i,j at $\tau_m = 0.2$ s		Inter-protons i,j at $\tau_m = 0.5$ s	
$\mid i - j \mid = 1$	2	$\mid i - j \mid = 1$	2
$\mid i - j \mid = 2$	1	$\mid i - j \mid = 2$	1
$\mid i - j \mid = 3$	2	$\mid i - j \mid = 3$	2
$\mid i - j \mid = 4$	3	$\mid i - j \mid = 4$	3
$\mid i - j \mid = 5$	4	$\mid i - j \mid = 5$	4
$\mid i - j \mid = 6$	8	$\mid i - j \mid = 6$	7
$\mid i - j \mid = 7$	6	$\mid i - j \mid = 7$	6
$\mid i - j \mid = 7.5$	1	$\mid i - j \mid = 8$	7
$\mid i - j \mid = 8$	7	$\mid i - j \mid = 9$	5
$\mid i - j \mid = 8.5$	1	$\mid i - j \mid = 10$	4
$\mid i - j \mid = 9$	5	$\mid i - j \mid = 11$	3
$\mid i - j \mid = 10$	4	$\mid i - j \mid = 12$	2
$\mid i - j \mid = 11$	2	$\mid i - j \mid = 13$	3
$\mid i - j \mid = 12$	1	$\mid i - j \mid = 15$	1
$\mid i - j \mid = 13$	1		
Total	48	Total	50

**Structural statistics**	
Number of NOE violations > 0.5 Å	0
Number of NOE violations > 0.2 Å	0
Number of NOE violations > 0.1 Å	0
Mean global rms	0.59 ± 0.49 (1.16 ± 1.07) (Å)
**Deviation from idealized geometry**	
Mean rms bond	$4.75\times10^{-3} \pm 1.7\times10^{-4}$ ($5.1\times10^{-3} \pm 6.1\times10^{-4}$) (Å)
Mean rms angle	$0.91 \pm 7.0\times10^{-3}$ ($0.92 \pm 2.6\times10^{-2}$) (degrees)
Mean rms improper	$1.55 \pm \times10^{-2}$ ($1.55 \pm 1.1\times10^{-2}$) (degrees)
Mean rms dihedral	$0.66 \pm 6.5\times10^{-3}$ ($0.66 \pm 6.2\times10^{-3}$) (degrees)
**Mean energies (kcal.mol^{-1})**	
E_{bonds}	1.18 ± 0.09 (1.37 ± 0.34)
E_{angles}	11.68 ± 0.18 (12.06 ± 0.69)
$E_{impropers}$	11.01 ± 0.14 (10.96 ± 0.16)
$E_{dihedrals}$	$3.60 \pm 7.1\times10^{-2}$ ($3.61 \pm 6.8\times10^{-2}$)
E_{vdw}	–11.76 ± 0.68 (–11.37 ± 0.88)
E_{total}	15.72 ± 0.68 (16.62 ± 1.69)

Table S10. Related to Figure 5. Summary of structural constraints and structure statistics for a set of 15 structures of LTB4 associated to human BLT2 receptor. In parenthesis are indicated statistics for an ensemble of 20 structures for which we also included structures 16 to 20 which display a total energy of ∼2 to 4 kcal/mol higher than the other structures (see also some structural features regarding these 5 additional structures in Figure S10).

HADDOCK score	-42.3 ± 1.6
Cluster size	193/200
RMSD from the overall lowest-energy structure (kcal/Mol)	0.6 ± 0.4
Van der Waals energy (kcal/mol)	-20.6 ± 2.0
Electrostatic energy (kcal/mol)	-65.7 ± 36.2
Desolvation energy (kcal/mol)	-8.7 ± 7.5
Restraints violation energy (kcal/mol)	0.1 ± 0.12
Buried Surface Area (Å^2)	678.1 ± 20.5
Z-Score	0.0

Table S11. Related to Figure 4. HADDOCK structural statistics. Two active residues in the receptor were used: S174 and R270. The passive residues were automatically defined around the active residues by HADDOCK. These statistics concern a docking simulation that generated only one cluster.

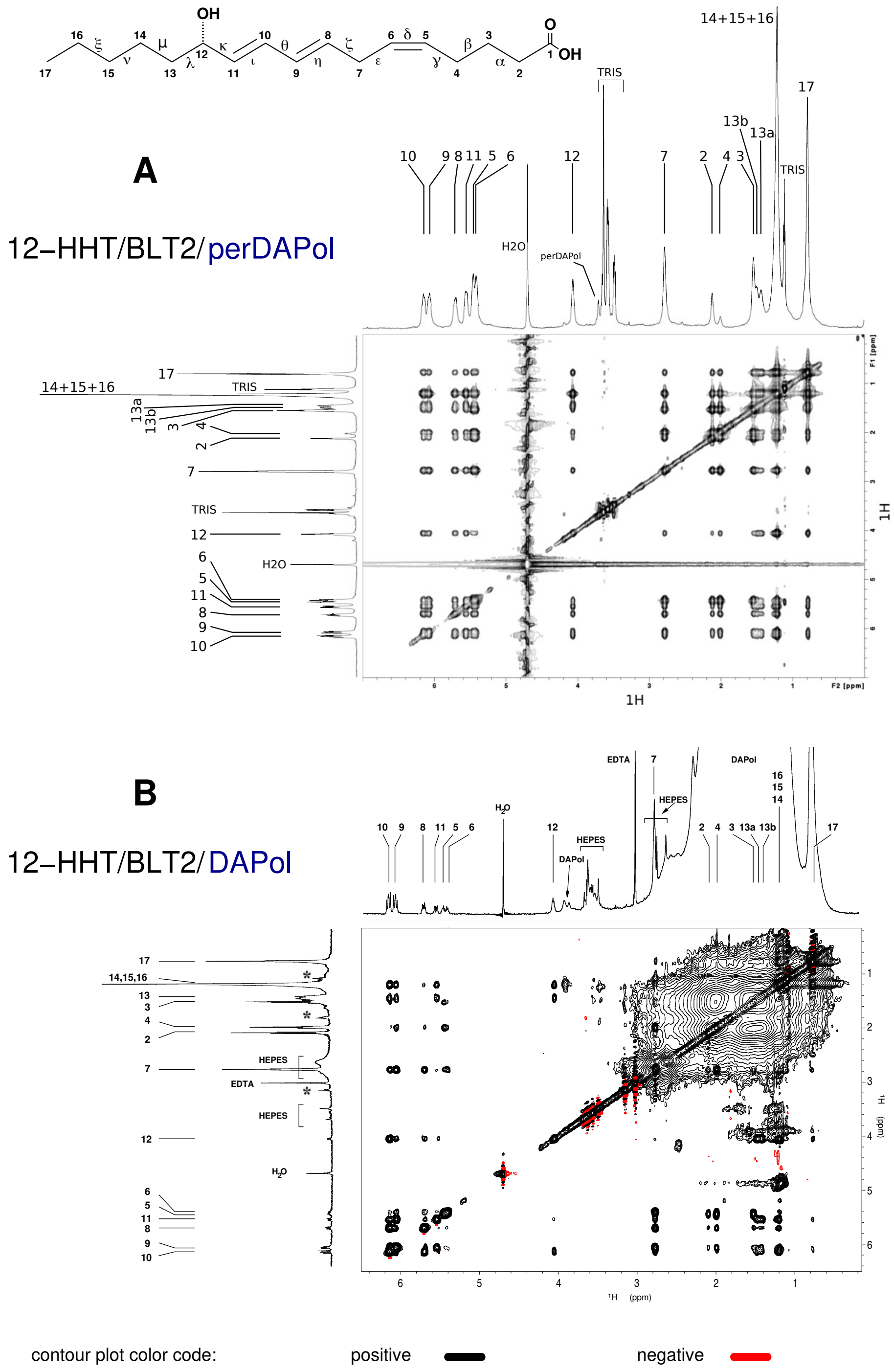

FIGURE S1. Related to Figure 1. Comparative 2D ^{1}H,^{1}H NOESY experiments to highlight the observation of intra-aliphatic ligand dipolar interactions thanks to the use of a perdeuterated amphipol. (**A**) The present study: NMR experiment showing intra-12–HHT dipolar interactions in the presence of BLT2 receptor associated to a perdeuterated amphipol, perDAPol (NOE mixing time $\tau_m = 0.5$ s; ^{1}H Larmor frequency $\nu_H = 700$ MHz). (**B**) Same experiment performed with BLT2 associated to a partially deuterated amphipol named DAPol (NOE mixing time $\tau_m = 0.4$ s; ^{1}H Larmor frequency $\nu_H = 600$ MHz) (adapted from Catoire et al., 2011).

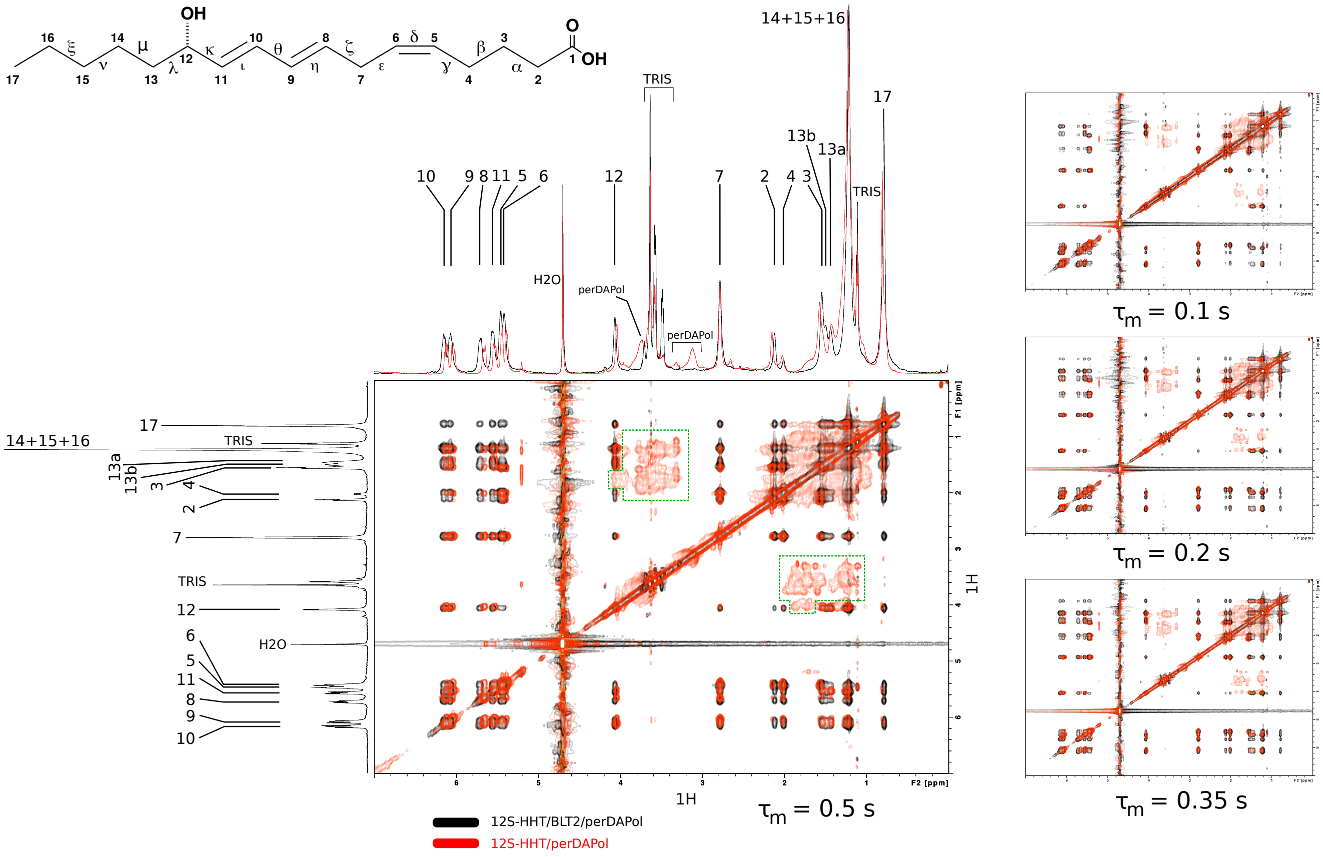

Figure S2. Related to Figure 1 and Figure 2. Superimposed 2D NOESY experiments of 12–HHT/perDAPol (in *red*) vs. 12–HHT/BLT2/ perDAPol (in *black*). The interaction of 12–HHT with the polymer is observed in the presence of perDAPol only (essentially in the two symmetrical regions squared with a green dashed line in the spectrum plotted in *red*.) The volume of the cross peaks observed for protons located at both extremities of the ligand are negligeable compared to those observed in the presence of the receptor, indicating a non-structured conformation at both ends of the ligand in the absence of the protein. This is particularly visible here for the rows that correspond to the chemical shifts of spins H2 and H17 (see also next Figure S3).

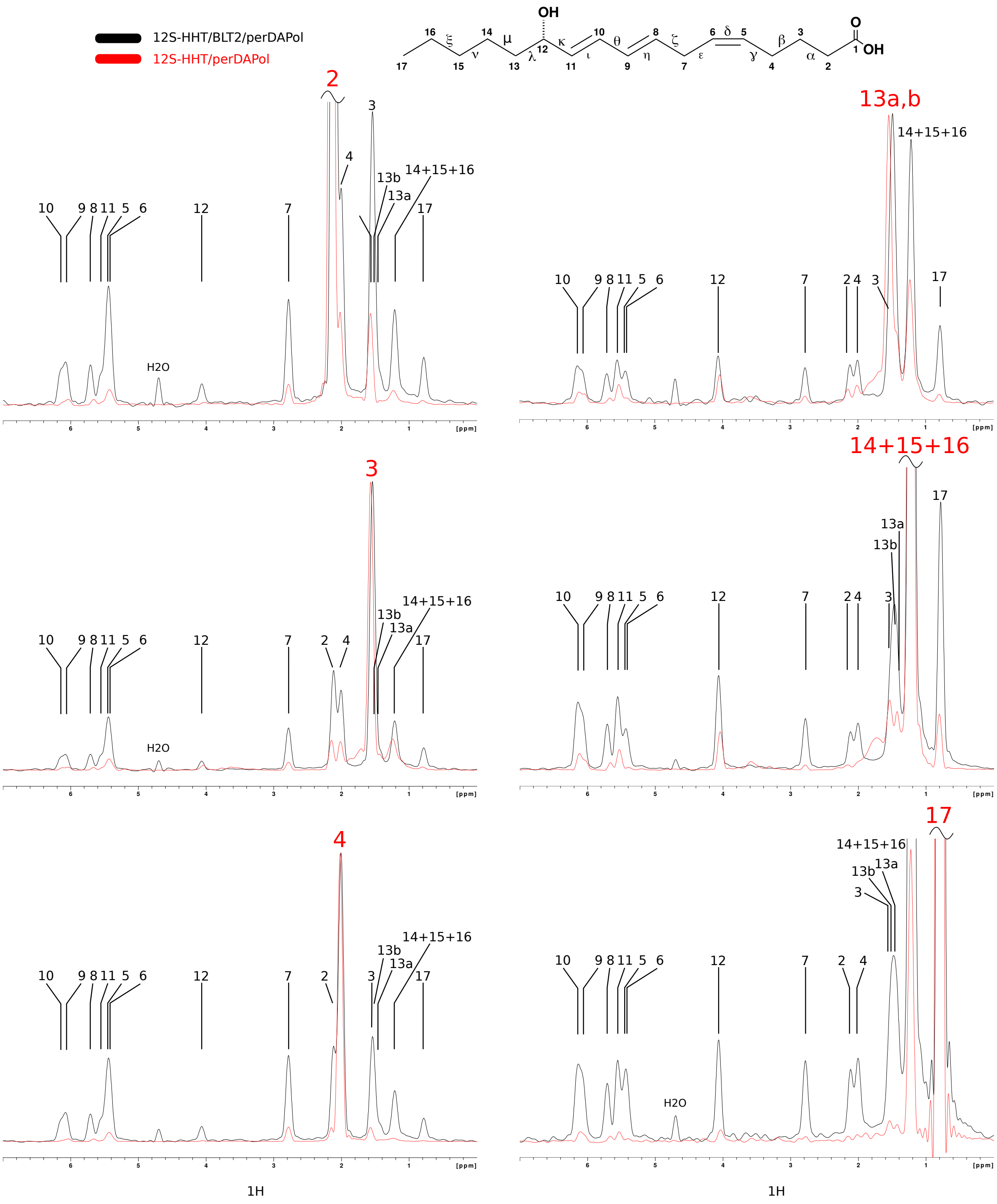

Figure S3. Related to Figure 1 and Figure 2. 1D columns extracted from the 2D NOESY spectrum displayed in Figure S2 of 12–HHT in the presence of wild-type receptor (in *black*) compared to columns extracted at the same frequency from a 2D NOESY spectrum obtained under the same conditions in the presence of perDAPol only (in *red*). All the columns have been normalized with respect to the diagonal peak (indicated by a red number).

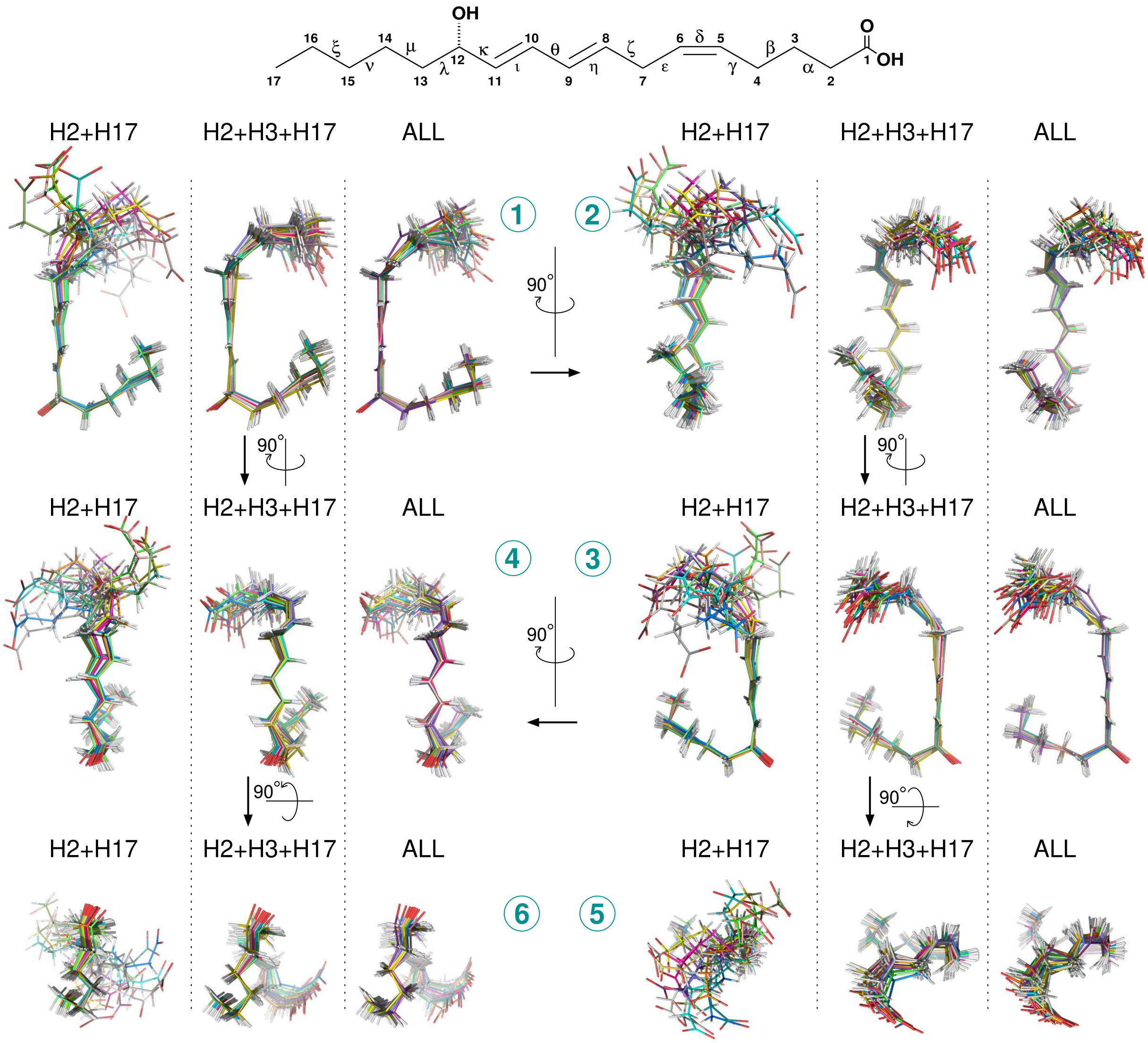

FIGURE S4. Related to Figure 2. Evolution of the structure calculations of 12–HHT by using an increasing number of NMR constraints. *All* correspond to all NMR constraints (which correspond to all the peaks except those in the boxes colored in *red* in Tables S1 to S4). By taking dipolar interactions involving spins H2, H3 and H17 only – with the rest of the protons in the molecule– this is enough to get a converged set of structures almost identical to the set of structures obtained by using all the restraints.

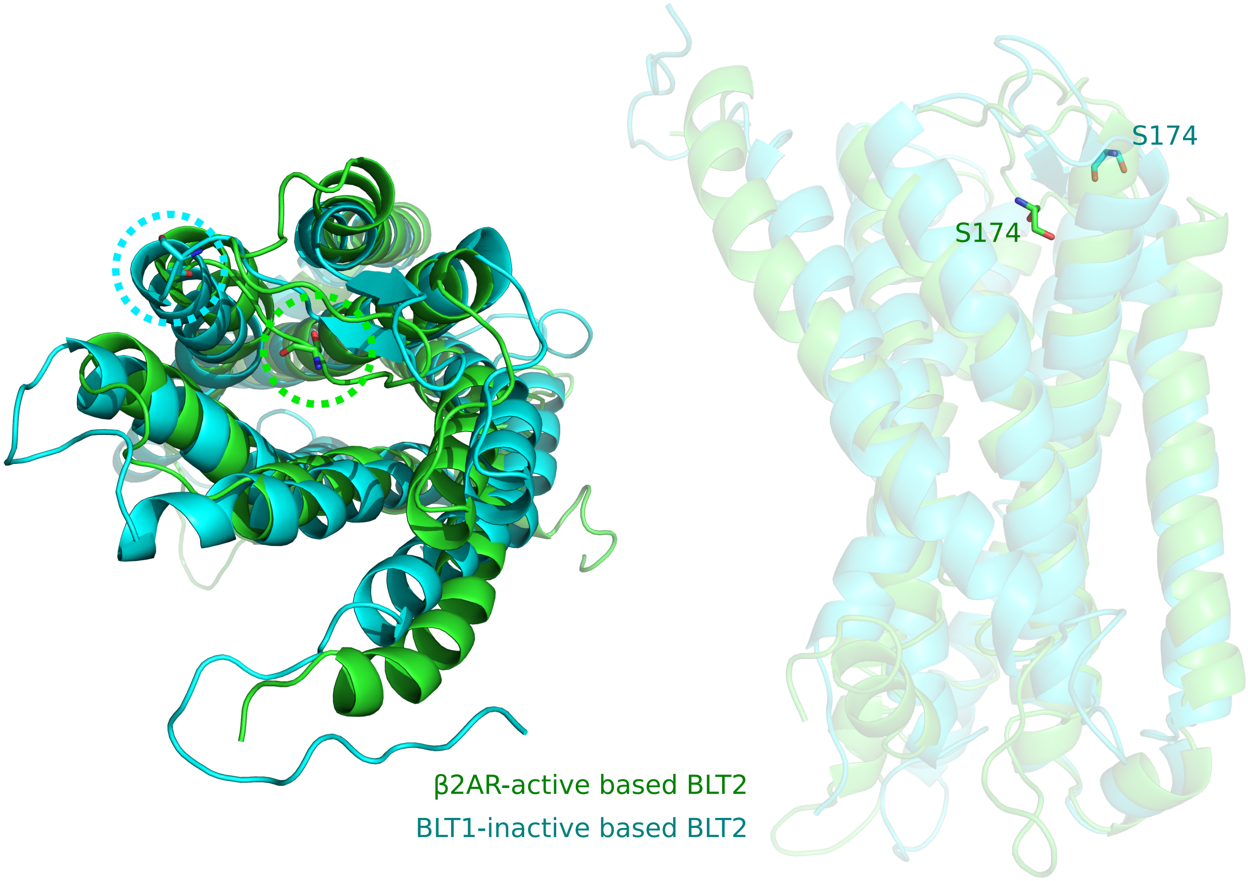

Figure S5. Related to Figure 4. Model comparison of human BLT2 based on the active state of the β2AR (in *green*) and an inactive state of BLT1 receptor (in *cyan*). On the *left*, a view from the extracellular side on an axe perpendicular to the membrane plane which indicates the position of the residue S174 (circled with a dotted line in both models) which mutation has a strong impact on 12–HHT binding (Figure 4). On the *right*, the two superimposed models are represented along an axis parallel to the membrane. From this side-view, in the case of β2AR-active state based BLT2 model, the extra-cellular loop 2 is located above the ligand orthosteric pocket in contrast with BLT1-based BLT2 model. The cartoon representation has been prepared with PyMOL software.

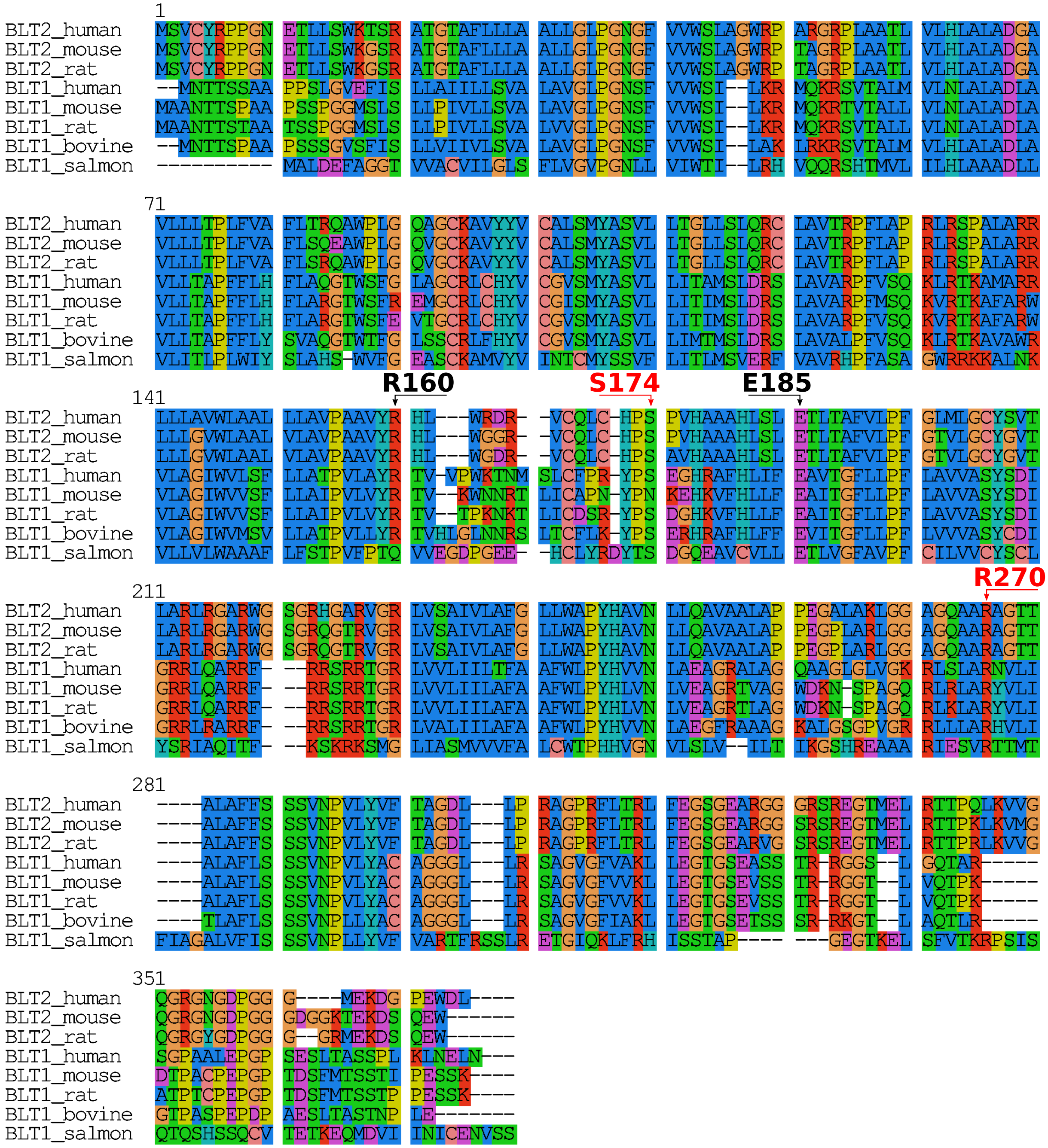

Figure S6. Related to Figure 4. Amino acid sequence alignments between BLT1 and BLT2 (SeaView software, Gouy et al., 2010). Above the alignments are indicated in *red* and *black* important residues in 12–HHT binding to BLT2 and LTB4 binding to BLT1, respectively.

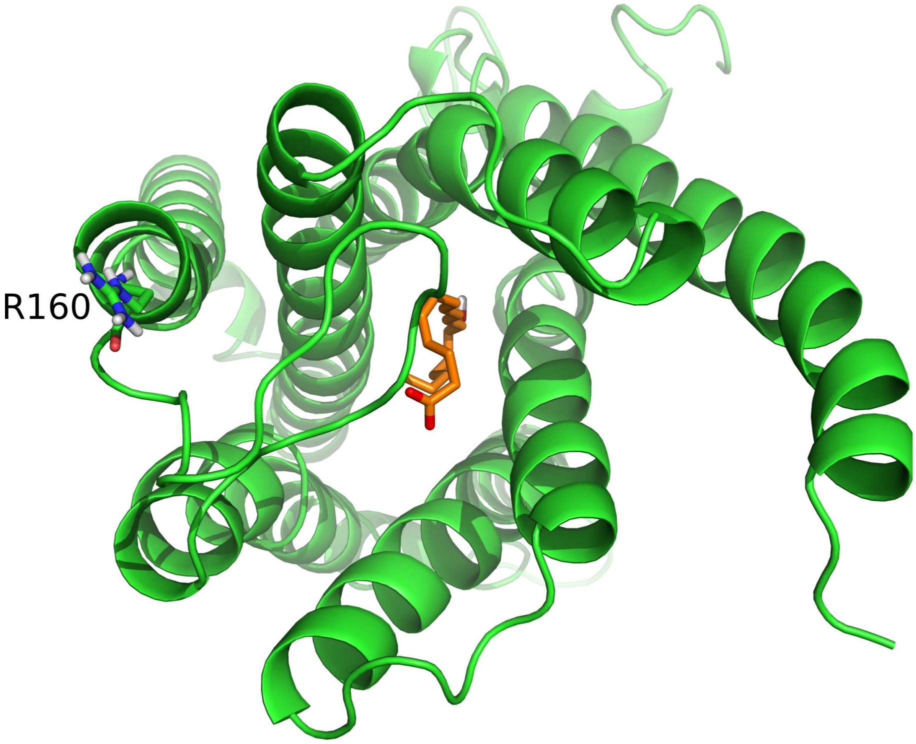

Figure S7. Related to Figure 4. Localization of R160$^{4.64}$ ($\equiv$ R156$^{4.64}$ in BLT1) in β2AR-active-based BLT2 model respectively to 12–HHT (in *orange*). The mutation of this residue in BLT1 results in a complete loss of LTB4 binding (Basu et al., 2007) (this representation has been performed in PyMOL).

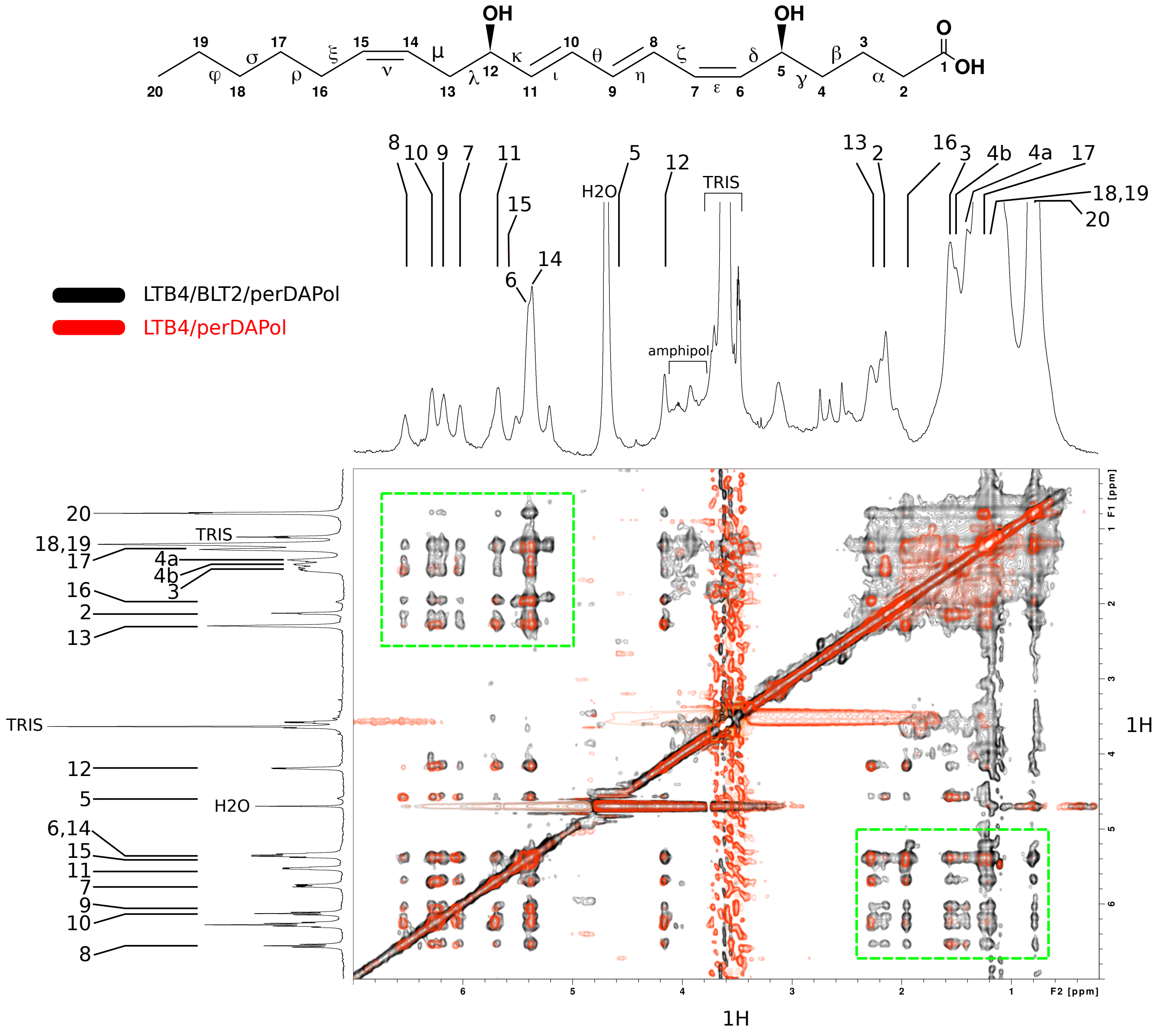

FIGURE S8. Related to Figure 5. Intra-LTB4 dipolar ^1H–^1H interactions observed in the presence of BLT2 associated to perDAPol in solution (in *black*; illustration with a 2D NOESY experiment acquired with a τ_m mixing time of 0.5 s). In *red*, a 2D NOESY experiment of LTB4 in the presence of perDAPol only ($\tau_m = 0.5$ s). Most of the volumes of the peaks involving aliphatic protons in the red spectrum (regions squared with a dashed green line) are negligeable compared to those observed in the presence of the receptor, indicating a non-structured conformation at both ends of the ligand in the absence of the protein.

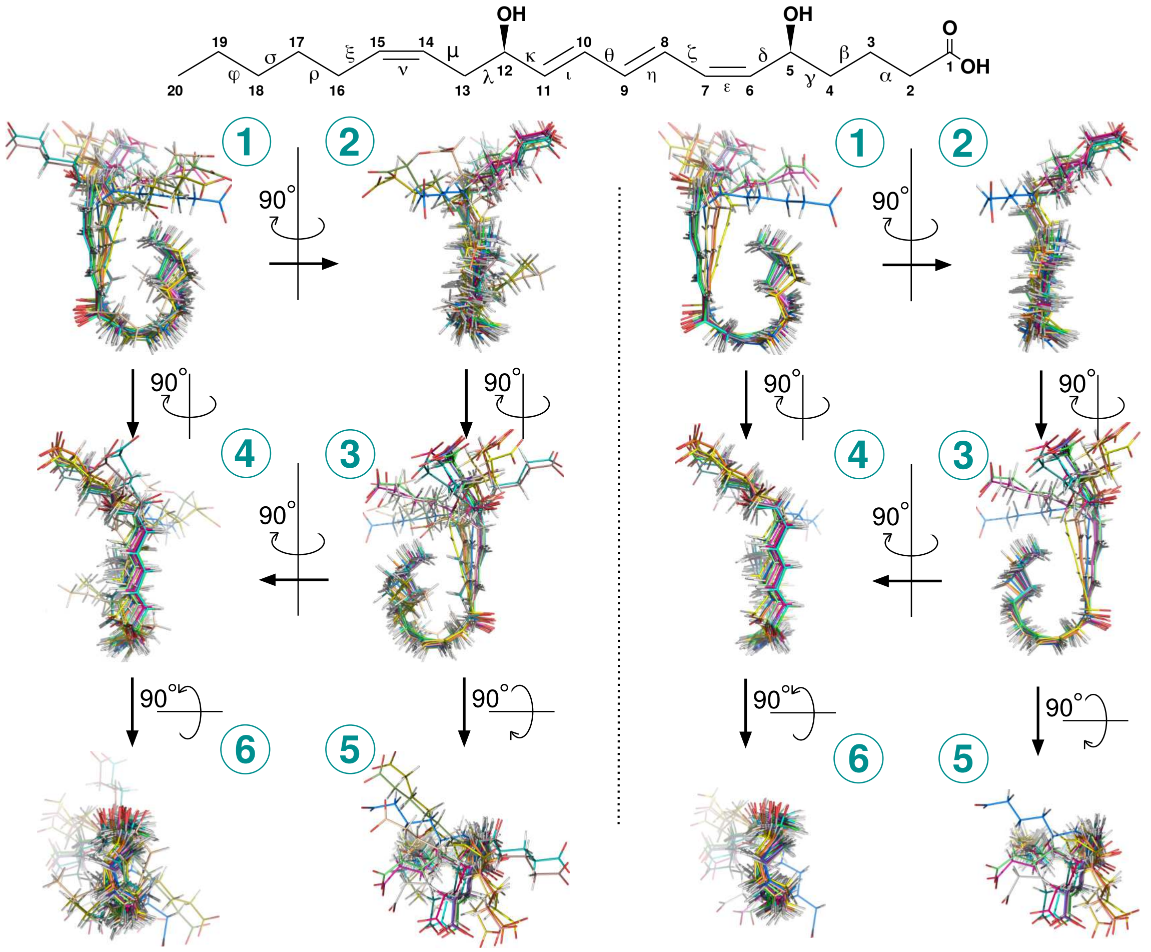

FIGURE S9. Related to Figure 5. Three-dimensional structure of LTB4 associated to BLT2 illustrated with six different views of one ensemble of 20 energy-minimized (on the *left*) or 15 energy-minimized (on the *right*) conformers, *i.e.* without the five highest energy structures listed in *red* in Table S10 (in white, hydrogen atoms; in red, oxygen atoms; carbon atoms are assigned a different color for each conformer). On *top* is displayed the primary chemical structure of LTB4. The carbons are numbered from the carboxyl function to the methyl group. Greek letters refer to some dihedral angles displayed in the next Figure S10 (the structures represented have been created using PyMOL).

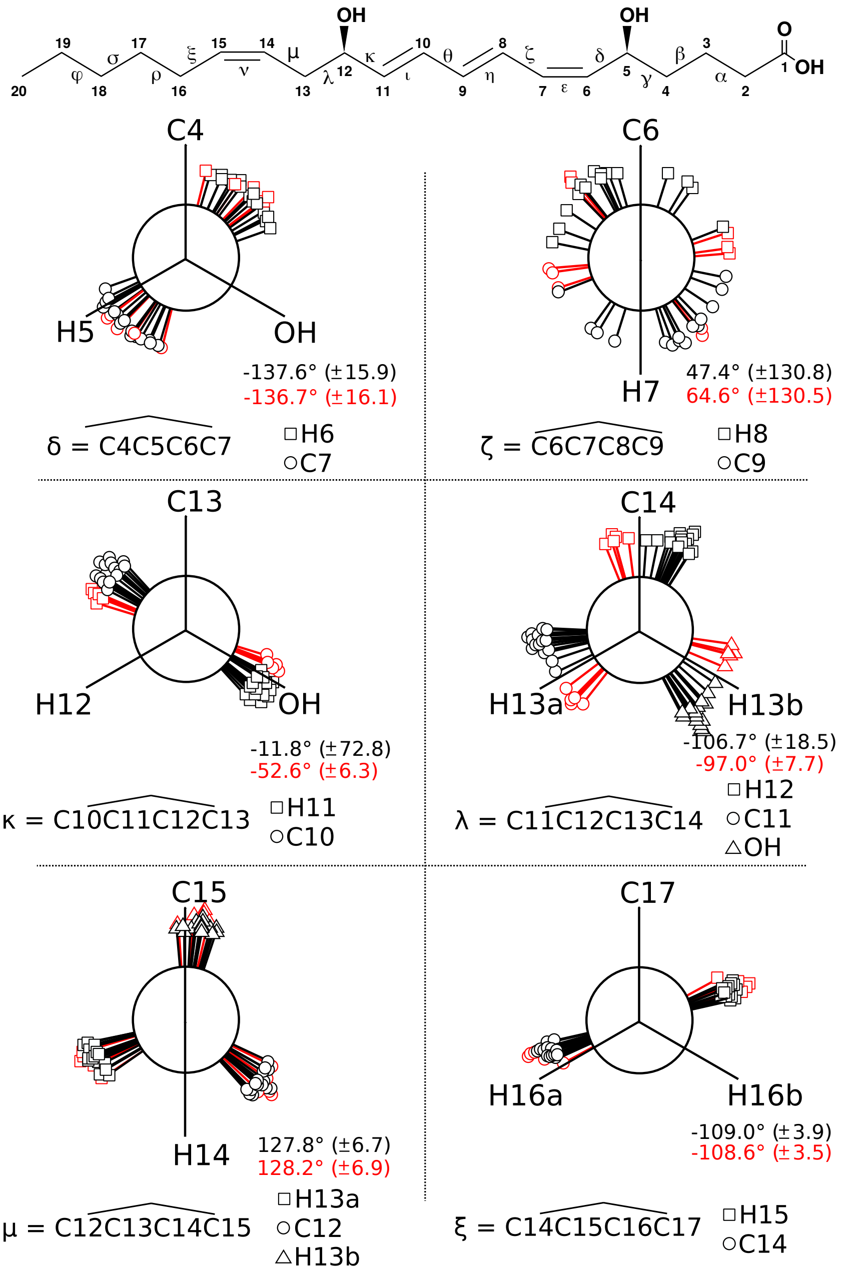

Figure S10. Related to Figure 5. Dihedral angles from the ensemble of 20 structures of LTB4 displayed in Figure S9. Data and dihedral represention in *red* correspond to the 5 highest energy conformers in this ensemble (see also Table S10).

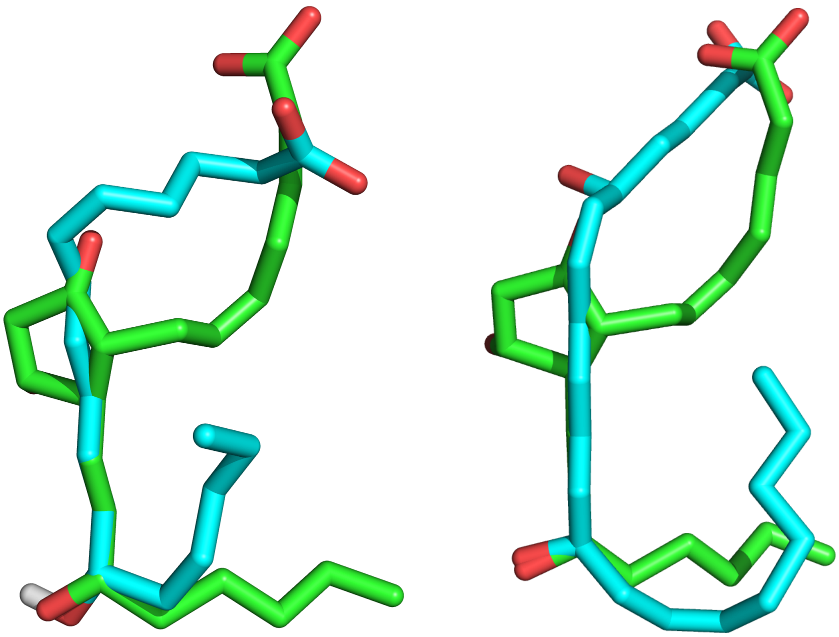

FIGURE S11. Superimpositions of one NMR conformer of 12–HHT (*right*) and LTB4 (*left*) bound to BLT2 on the crystal structure of the eicosanoid prostaglandin E2 bound to EP3 receptor (Morimoto et al., 2019; pdb id = 6AK3).

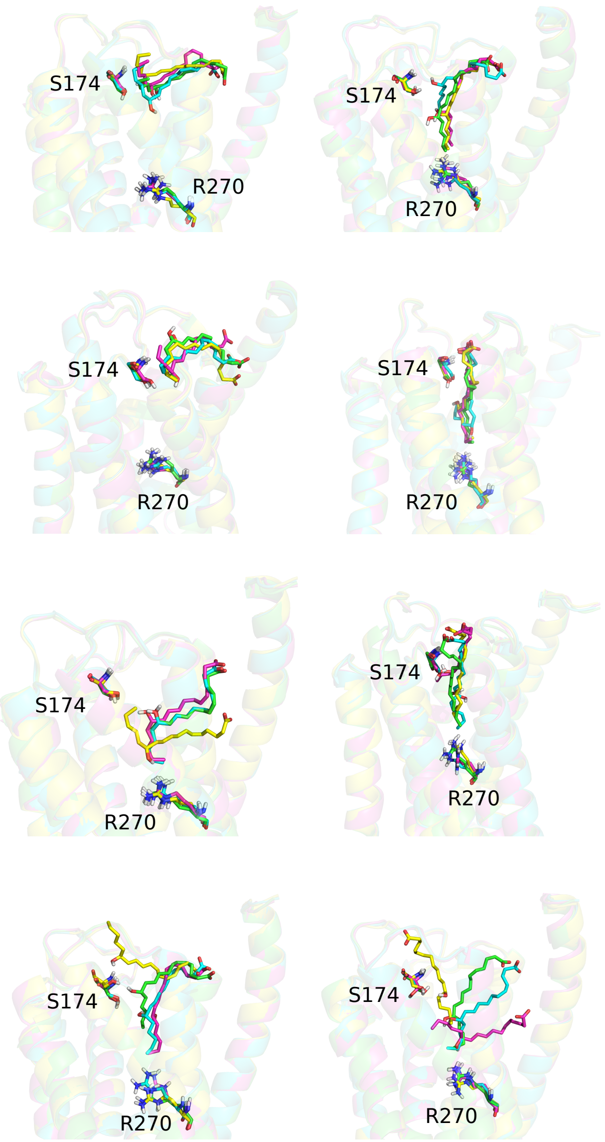

FIGURE S12. Docking simulations of the set of 20 12–HHT conformers free in solution (from Figure 2). Illustration with 8 conformers. For each conformer, only the best cluster composed of four models was represented. None of the 20 simulations could display hydrogen bonds between the ligand and S174 and R270 at the same time.

References

[Basu et al., 2007] Basu, S., Jala, V.R., Mathis, S., Rajagopal, S.T., Del Prete, A., Maturu, P., Trent, J.O., and Haribabu, B. (2007) Critical role for polar residues in coupling leukotriene B4 binding to signal transduction in BLT1. J. Biol. Chem. *282*, 10005-10017.

[Catoire et al., 2011] Catoire, L.J., Damian, M., Baaden, M., Guittet, E., and Banères J.L. (2011) Electrostatically-driven fast association and perdeuteration allow detection of transferred cross-relaxation for G protein-coupled receptor ligands with equilibrium dissociation constants in the high-to-low nanomolar range. J. Biomol. NMR. *50*, 191-195.

[Gouy et al., 2010] Gouy, M., Guindon, S., and Gascuel, O. (2010) SeaView version 4 : a multiplatform graphical user interface for sequence alignment and phylogenetic tree building. Mol. Biol. Evol. *27*, 221-224.

[Morimoto et al., 2019] Morimoto, K., Suno, R., Hotta, Y., Yamashita, K., Hirata, K., Yamamoto, M., Narumiya, S., Iwata, S., Kobayashi, T. (2019) Crystal structure of the endogenous agonist-bound prostanoid receptor EP3. Nat. Chem. Biol. *15*, 8-10.
